# Neurocircuitry underlying the antidepressant effect of retrograde facial botulinum toxin in mice

**DOI:** 10.1186/s13578-023-00964-1

**Published:** 2023-02-13

**Authors:** Linhui Ni, Hanze Chen, Xinxin Xu, Di Sun, Huaying Cai, Li Wang, Qiwen Tang, Yonggang Hao, Shuxia Cao, Xingyue Hu

**Affiliations:** 1 Department of Neurology, Sir Run Run Shaw Hospital, School of Medicine, Zhejiang University, Hangzhou, 310053 China; 2 Department of Ultrasonography, Sir Run Run Shaw Hospital, School of Medicine, Zhejiang University, Hangzhou, 310053 China; 3 Department of Neurology, Dushu Lake Hospital Affiliated to Soochow University, Suzhou, 215125 China

**Keywords:** Botulinum toxin type A (BoNT/A), Antidepressant, Neurocircuitry, Retrograde transport, Whisker-innervating facial motoneurons (wFMNs), Ventrolateral periaqueductal grey (vlPAG)

## Abstract

**Backgrounds:**

Botulinum toxin type A (BoNT/A) is extensively applied in spasticity and dystonia as it cleaves synaptosome-associated protein 25 (SNAP25) in the presynaptic terminals, thereby inhibiting neurotransmission. An increasing number of randomized clinical trials have suggested that glabellar BoNT/A injection improves depressive symptoms in patients with major depressive disorder (MDD). However, the underlying neuronal circuitry of BoNT/A-regulated depression remains largely uncharacterized.

**Results:**

Here, we modeled MDD using mice subjected to chronic restraint stress (CRS). By pre-injecting BoNT/A into the unilateral whisker intrinsic musculature (WIM), and performing behavioral testing, we showed that pre-injection of BoNT/A attenuated despair- and anhedonia-like phenotypes in CRS mice. By applying immunostaining of BoNT/A-cleaved SNAP25 (cl.SNAP25_197_), subcellular spatial localization of SNAP25 with markers of cholinergic neurons (ChAT) and post-synaptic membrane (PSD95), and injection of monosynaptic retrograde tracer CTB-488-mixed BoNT/A to label the primary nucleus of the WIM, we demonstrated that BoNT/A axonal retrograde transported to the soma of whisker-innervating facial motoneurons (wFMNs) and subsequent transcytosis to synaptic terminals of second-order neurons induced central effects. Furthermore, using transsynaptic retrograde and monosynaptic antegrade viral neural circuit tracing with c-Fos brain mapping and co-staining of neural markers, we observed that the CRS-induced expression of c-Fos and CaMKII double-positive neurons in the ventrolateral periaqueductal grey (vlPAG), which sent afferents to wFMNs, was down-regulated 3 weeks after BoNT/A facial pre-administration. Strikingly, the repeated and targeted silencing of the wFMNs-projecting CaMKII-positive neurons in vlPAG with a chemogenetic approach via stereotactic injection of recombinant adeno-associated virus into specific brain regions of CRS mice mimicked the antidepressant-like action of BoNT/A pre-treatment. Conversely, repeated chemogenetic activation of this potential subpopulation counteracted the BoNT/A-improved significant antidepressant behavior.

**Conclusion:**

We reported for the first time that BoNT/A inhibited the wFMNs-projecting vlPAG excitatory neurons through axonal retrograde transport and cell-to-cell transcytosis from the injected location of the WIM to regulate depressive-like phenotypes of CRS mice. For the limited and the reversibility of side effects, BoNT/A has substantial advantages and potential application in MDD.

**Supplementary Information:**

The online version contains supplementary material available at 10.1186/s13578-023-00964-1.

## Introduction

Major depressive disorder (MDD), a mental disorder characterized by anhedonia, low mood, loss of motivation, and despair, affects more than 300 million people worldwide, with a global cumulative incidence of approximately 4.4% until 2017 [1]. MDD negatively impacts human health and economic development [2, 3]. Current antidepressants include selective serotonin reuptake inhibitors, norepinephrine reuptake inhibitors, and tricyclic antidepressants, which target the monoamine system. However, up to 20% of patients with MDD do not respond to existing antidepressants [4], which can also be associated with delayed therapeutic response and negative side effects. Therefore, improved therapeutic strategies are urgently needed.

Botulinum toxin type A (BoNT/A), a potent toxin produced by *Clostridium botulinum*, is utilized to treat movement disorders and neuropathic pain, and for cosmetic purposes. BoNT/A reversibly blocks the release of neurotransmitters through the cleavage of synaptosome-associated protein 25 (SNAP25) in the synaptic terminals [5]. Its enzymatic activity was traditionally considered to be limited to local injection loci. However, distant and central effects following peripheral application of BoNT/A have been identified. Central reorganization was found in patients with dystonia treated with BoNT/A. For example, functional magnetic resonance imaging (fMRI) in patients with Meige’s syndrome post-BoNT/A injection showed reduced activation in the left supplementary motor cortex, bilateral thalami, and contralateral putamen [6]. Moreover, increasing evidence reveals that BoNT/A can bypass neuronal connections via retrogradely transport and transcytosis from the periphery to the central nervous system (CNS) [7–9].

An increasing number of randomized controlled trials have shown that BoNT/A injected into the corrugator and musculus procerus relieves depressive symptoms in patients with MDD [10–13]. Three major theories have been proposed to explain this antidepressant efficacy [14]. First, cosmetic effectiveness leads to pleasant facial expressions, encouraging positive social feedback [14]. Second, the relieved facial muscles prevent negative facial expressions and alter the afferent nerve signals to the CNS [15, 16]. Third, BoNT/A might directly affect emotion processing via retrograde transport through neuroanatomical circuitry to related brain regions [7–9]. In preclinical research, Li et al. reported that mice with spatial restraint stress-induced depression exhibited antidepressant-like behavioral effects 1 h after bilateral BoNT/A facial injection that continued for at least 7 days, together with increased serotonin and brain-derived neurotrophic factor (BDNF) levels and activation of the BDNF–ERK–CREB pathway in the hippocampus [17]. Another study found that serotonin and noradrenaline were up-regulated in the hypothalamus and striatum of naïve rats following BoNT/A unilateral vibrissal injection [18]. Moreover, our previous research observed that unilateral facial BoNT/A application enhanced the learning ability of naïve mice 6 weeks post-injection, using a Morris water maze test [19]. Along with few reported cosmetic actions in rodents, BoNT/A injection leads to whisker apraxia, which is detrimental for social behavior and might negatively affect rodent emotion [20, 21], reflecting complex central mechanisms involved in the action of facial BoNT/A.

The dysfunction of some whisker-projecting premotor nuclei has been implicated in depression. As a columnar organization in the midbrain connected with some premotor nuclei in the hindbrain [22], including whisker-innervating facial motoneurons (wFMNs) [23, 24], the periaqueductal grey (PAG) also integrates input from the prefrontal cortex (PFC) [25, 26], central region of the amygdala (CeA) [26, 27], and hypothalamus [26, 28], which have been considered the pivotal elements of neuronal circuitry for regulating depressive performance. The PAG plays prominent roles in emotional processing, including pain processing [25, 27] defensive reactions [22, 30] fear and anxiety [57, 58] and stress-induced depression [29, 32, 59]. In the ventrolateral PAG (vlPAG), glutamatergic neurons, the largest excitatory neurons, mediate pain-induced depressive-like behavior in inflammatory bowel disease in a mouse model [31]. Additionally, the activation of GluR1 in the vlPAG leads to antidepressant effects in rats experiencing unpredictable foot shock stress [32]. Notably, wFMNs have been described to receive direct vlPAG inputs, as depicted by positive conjugated tag signals in the PAG following unilateral CTB injection into physiologically identified wFMNs [23], and using a modified transsynaptic rabies strategy to label the premotor areas of unilateral wFMNs [24]. Nevertheless, whether facially injected BoNT/A undergoes retrograde transport and transcytosis along such a potential neural pathway to participate in depressant regulation is poorly understood.

Here, we propose neurocircuitry underlying retrograde BoNT/A transport to regulate depression. Using a mouse model of chronic restraint sstress (CRS)-induced depression and drug administration together with behavioral testing, we determined whether facial injection of BoNT/A reversed the depressive-like behavior induced by CRS. Additionally, we used immunostaining, c-Fos brain mapping, neuroanatomical tracing, and specific chemogenetic manipulation to evaluate the contribution of the wFMNs-projecting vlPAG neurons to retrograde BoNT/A effects on depression. Together, our results shed new light on the direct antidepressant effect of BoNT/A facial injection via retrograde transport to the central nucleus related to emotion processing.

## Results

### Pre-injection of BoNT/A into whisker musculature alleviates depressive-like behaviors in CRS mice

**Fig. 1:**
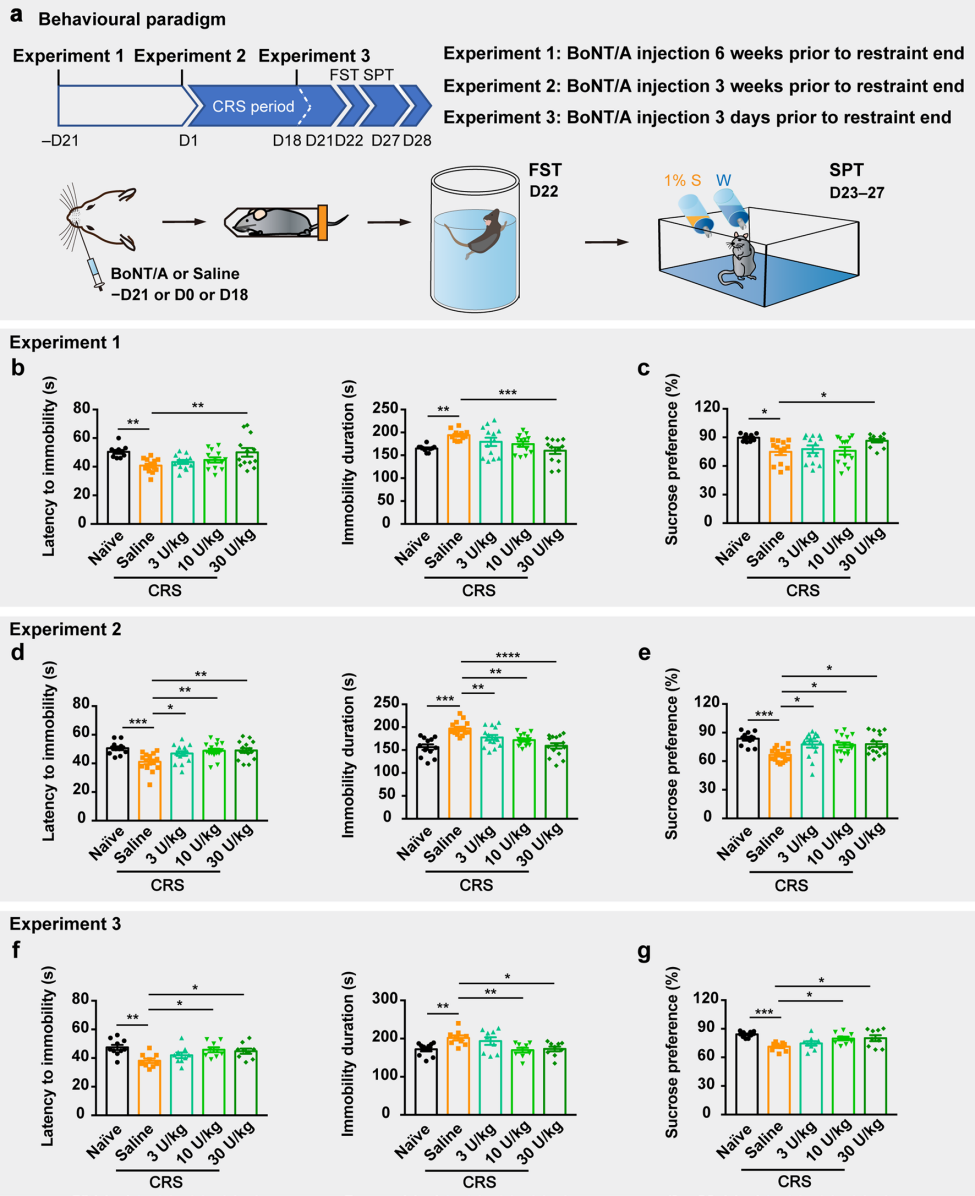
BoNT/A facial injection relieves depressive-like behaviors of CRS-induced depression mice. **a** Schematic of grouping and experimental design. **b**, **c** CRS mice performed depressive-like behaviors in the FST and SPT compared to the naïve mice, and 30 U/kg of BoNT/A facial pre-injection reverses the despair-, **b** and anhedonia-phenotypes, **c** of saline-administered CRS mice in the group of pre-injection 6 weeks prior to the restraint end. n = 10 animals from the subgroup of Naïve + Saline, and n = 13 animals from the other four subgroups, respectively. One-way ANOVA followed by Dunnett’s multiple comparisons test comparing each subgroup with the subgroup of CRS mice injected with saline, *F*_(4,__57)_ = 4.603, *P* = 0.0027 and *F*_(4,__57)_ = 4.65, *P* = 0.0026 in **b**; *F*_(4,__57)_ = 4.13, *P* = 0.0052 in **c**. The *P*-value of Dunnett’s multiple comparisons: Naïve + Saline *vs.* CRS + Saline: *P* = 0.0057, CRS + Saline *vs.* CRS + 3 U/kg: *P* = 0.7601, CRS + Saline *vs.* CRS + 10 U/kg: *P* = 0.3935, CRS + Saline *vs.* CRS + 30 U/kg: *P* = 0.004 of latency of immobility in **b**; Naïve + Saline *vs.* CRS + Saline: *P* = 0.0089, CRS + Saline *vs.* CRS + 3 U/kg: *P* = 0.2691, CRS + Saline *vs.* CRS + 10 U/kg: *P* = 0.0872, CRS + Saline *vs.* CRS + 30 U/kg: *P* = 0.0008 of total time of immobility in **b**; Naïve + Saline *vs.* CRS + Saline: *P* = 0.01, CRS + Saline *vs.* CRS + 3 U/kg: *P* = 0.9247, CRS + Saline *vs.* CRS + 10 U/kg: *P* = 0.9976, CRS + Saline *vs.* CRS + 30 U/kg: *P* = 0.0381 of sucrose preference in **c**. **d**, **e** CRS mice in the subgroups of facial pre-injection of three different dosages of BoNT/A performed relieved depressive-like behaviors depicted by improved despair and anhedonia in the group of BoNT/A pre-injection 3 weeks prior to the restraint end. n = 12 animals from the subgroup of Naïve + Saline, and n = 15 animals from the other four subgroups, respectively. One-way ANOVA followed by Dunnett’s multiple comparisons test comparing each subgroup with the subgroup of CRS mice injected with saline, *F*_(4,__67)_ = 3.364, *P* = 0.0144 and *F*_(4,__57)_ = 11, *P* < 0.0001 in **d**; *F *_(4,__57)_ = 5.241, *P* = 0.0010 in **e**. The *P*-value of Dunnett’s multiple comparisons: Naïve + Saline *vs.* CRS + Saline: *P* = 0.0004, CRS + Saline *vs.* CRS + 3 U/kg: *P* = 0.0307, CRS + Saline *vs.* CRS + 10 U/kg: *P* = 0.0023, CRS + Saline *vs.* CRS + 30 U/kg: *P* = 0.0017 of latency of immobility in **d**; Naïve + Saline *vs.* CRS + Saline: *P* = 0.0001, CRS + Saline *vs.* CRS + 3 U/kg: *P* = 0.0203, CRS + Saline *vs.* CRS + 10 U/kg: *P* = 0.0016, CRS + Saline *vs.* CRS + 30 U/kg: *P* = 0.0001 of total time of immobility in **d**; Naïve + Saline *vs.* CRS + Saline: *P* = 0.0002, CRS + Saline *vs.* CRS + 3 U/kg: *P* = 0.0147, CRS + Saline *vs.* CRS + 10 U/kg: *P* = 0.0206, CRS + Saline *vs*. CRS + 30 U/kg: *P* = 0.012 of sucrose preference in **e**. **f**, **g** CRS mice in the subgroups of 10 and 30 U/kg BoNT/A facial pre-injection exhibited attenuated depressive-like behaviors compared to that in the subgroup treated with saline in the group of facial pre-treatment 3 days prior to the restraint end. n = 10 animals from the subgroup of Naïve + Saline, and n = 9 animals from the other four subgroups, respectively. One-way ANOVA followed by Dunnett’s multiple comparisons test comparing each subgroup with the subgroup of CRS mice with saline injection, *F*_(4,__41)_ = 4.239, *P* = 0.0058 and *F*_(4,__41)_ = 4.353, *P* = 0.005 in **f**; *F*_(4,__41)_ = 6.176, *P* = 0.0006 in **g**. The *P*-value of Dunnett’s multiple comparisons: Naïve + Saline *vs.* CRS + Saline: *P* = 0.0021, CRS + Saline *vs.* CRS + 3 U/kg: *P* = 0.3919, CRS + Saline *vs.* CRS + 10 U/kg: *P* = 0.016, CRS + Saline *vs.* CRS + 30 U/kg: *P* = 0.0422 of latency of immobility in **f**; Naïve + Saline *vs.* CRS + Saline: *P* = 0.0137, CRS + Saline *vs.* CRS + 3 U/kg: *P* = 0.8188, CRS + Saline *vs.* CRS + 10 U/kg: *P* = 0.009, CRS + Saline *vs.* CRS + 30 U/kg: *P* = 0.0216 of total time of immobility in **f**; Naïve + Saline *vs.* CRS + Saline: *P* = 0.0002, CRS + Saline *vs.* CRS + 3 U/kg: *P* = 0.6062, CRS + Saline *vs.* CRS + 10 U/kg: *P* = 0.0205, CRS + Saline *vs.* CRS + 30 U/kg: *P* = 0.0146 of sucrose preference in **g**. *P < 0.05, **P < 0.01, ***P < 0.001, ****P < 0.0001

Repeated restraint stress leads to depressive-like behavioral phenotypes in rodents, representing a traditional model extensively used in depressive or anxiolytic studies [33]. Mice were restrained for 2–3 h per day for 21 consecutive days [27, 34], then the behaviors of despair and anhedonia were tested via FST and SPT. We successfully constructed the depression model of CRS mice as demonstrated by decreased latency to immobility and increased duration thereof in the FST, and decreased preference for sucrose in the SPT (Fig. 1).

To investigate the antidepressant effects of BoNT/A facial injection, CRS mice were pre-injected with three dosages of BoNT/A or saline into the unilateral whisker intrinsic musculature (WIM) at three different time points prior to the end of the restraint period (6 weeks, 3 weeks and 3 days before 21-day restraint stress end, Fig. 1a). After the 21-day restraint stress terminated, the FST and SPT were done to test the depressive-like behavior of mice from different subgroups. In CRS mice that were administered BoNT/A 6 weeks prior to the restraint end (Experiment 1, equivalently to 3 weeks prior to the restraint start), only the subgroup receiving 30 U/kg of BoNT/A exhibited longer latency to immobility, shorter immobility duration in the FST (Fig. 1b), and increased sucrose preference in the SPT (Fig. 1c), compared to the performance of CRS mice that received saline. CRS mice in the subgroups pre-treated with three different doses of BoNT/A 3 weeks prior to the end of restraint (Experiment 2, equivalently to 1 day prior to the restraint start) also demonstrated increased latency to immobility and decreased immobility duration in the FST (Fig. 1d), with increased sucrose preference in the SPT (Fig. 1e), compared with the performance of saline-treated CRS animals. In addition, in the group injected with BoNT/A 3 days prior to the end of the 21-day-CRS treatment (Experiment 3), CRS mice in the 10 and 30 U/kg subgroups exhibited increased latency to immobility and reduced immobility duration in the FST (Fig. 1f), as well as enhanced sucrose preference (Fig. 1g), however, the performances of CRS mice injected with 3 U/kg of BoNT/A were in no difference from the CRS mice injected with saline (Fig. 1f–g). Furthermore, three BoNT/A doses resulted in flaccid paralysis of the injected musculature 24 h post-injection (Additional file 2: Fig. S1b–d). While whisker movement recovered 2–3 weeks later, the antidepressant effect remained for a substantially longer period.

To exclude the possibility that unilateral BoNT/A facial injection might impair locomotor activity, which might, in turn, interfere with behavioral states as measured by FST or SPT, the movement ability of all mice was tested using the OFT subsequent to FST and SPT (Additional file 2: Fig. S2a). The total distance travelled, mean speed, and time in the center zone of saline-administrated CRS mice in the OFT were not different from those of naïve mice or BoNT/A-administrated CRS mice (Additional file 2: Fig. S2b–d).

### BoNT/A transsynaptic enters second-order projecting neuron boutons after axonal retrograde transports to wFMNs soma

**Fig. 2:**
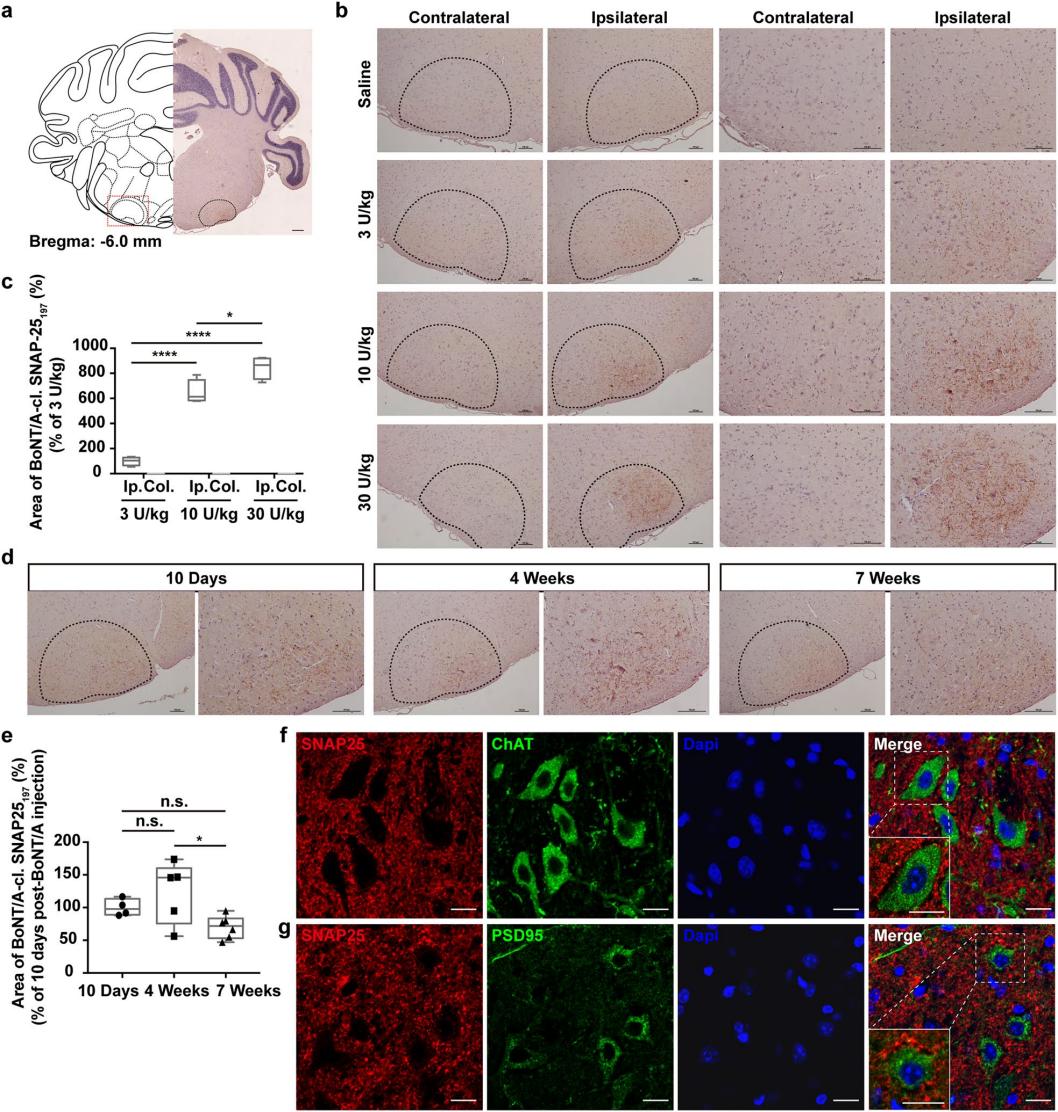
BoNT/A enzymatically cleaves SNAP25 in wFMNs-projecting synaptic terminals. **a** General schematic view of BoNT/A-cleaved SNAP25_197_ expression. **b** BoNT/A-cleaved SNAP25_197_ was detected on the ipsilateral lFN to BoNT/A injection side in subgroups of three different dosages 10 days post-BoNT/A WIM injection. Right two lines, magnified view of the left two lines. Scale bar, 100 μm. **c** Quantificational analysis of BoNT/A-cleaved SNAP25_197_ among subgroups of three different BoNT/A dosages. *n* = 4 cells from 3 mice per subgroup, respectively. One-way ANOVA followed by Bonferroni’s multiple comparisons test, *F*_(2,__9)_ = 99.68, *P* < 0.0001. Bonferroni’s multiple comparisons: 3 U/kg *vs.* 10 U/kg: *P* < 0.0001, 3 U/kg *vs.* 30 U/kg: *P* < 0.0001, 10 U/kg vs. 30 U/kg: *P* = 0.0169. Intensity of the immunostaining for each subgroup was normalized to the labelling level at the subgroup of 3 U/kg. **d** Representative images of cl.SNAP25_197_ expression on lFN of three time points post-BoNT/A (10 U/kg) WIM pre-injection. The right image was the magnified view of the left one. Scale bar, 100 μm. **e** Quantificational analysis of the percentage of BoNT/A-cleaved SNAP25_197_-positive signal areas among groups of three time points post-BoNT/A (10 U/kg) pre-injection, indicating that cl.SNAP25_197_ was detected at 10 days, with a highest expression at 4 weeks, and existed persistently to at least 7 weeks post-BoNT/A WIM injection. n = 4 brain sections from 3 mice in the group of 10 days, n = 5 brain sections from 3 mice in the group of 4 weeks, and n = 6 brain sections from 3 mice in the group of 7 weeks, respectively. One-way ANOVA followed by Bonferroni’s multiple comparisons test, *F*_(2,__12)_ = 4.33, *P* = 0.0384. The *P*-value of Bonferroni’s multiple comparisons: 10 Days *vs.* 4 Weeks: *P* = 0.8069, 10 Days *vs.* 7 Weeks: *P* = 0.4491, 4 Weeks *vs.* 7 Weeks: *P* = 0.0382. Intensity of the immunostaining for each group was normalized to the labeling level at the group of 10 days post-BoNT/A pre-injection. **f** Co-immunostaining of SNAP25 with ChAT showed non-colocalization of the two proteins. Scale bar, 50 μm. **g** Co-immunostaining of SNAP25 and PSD95 indicated non-colocalization. Scale bar, 50 μm. *P < 0.05, ****P < 0.0001, n.s., non-significance; Ip., ipsilateral side of BoNT/A injection side; Col., contralateral side of BoNT/A injection location

BoNT/A can be transported to the CNS from the periphery via retrograde axoplasmic transport and exocytosis [7–9]. To investigate the characteristics of retro-transsynaptic action and dose- or time-dependence of BoNT/A-mediated cleavage, we immunostained BoNT/A-cleaved SNAP25 (cl.SNAP25_197_) in hindbrain sections following WIM injection of BoNT/A. We detected cl.SNAP25_197_ only on one side of the lateral subnucleus of facial nucleus (lFN) (Fig. 2a–b), the location of intrinsic wFMNs, whereas cl.SNAP25_197_ was detected more dorsally in the FN in the subgroup administered 30 U/kg BoNT/A; however, no positive signals were detected in adjacent regions or the second-order nucleus of the three BoNT/A-subgroups (Fig. 2b and Additional file 2: Fig. S3). Notably, cl.SNAP25_197_ was not detected in the principal sensory trigeminal nucleus (Pr5) or the spinal trigeminal nucleus (Sp5) (Additional file 2: Fig. S3), which have been reported to receive afferent synapses from the trigeminal ganglion [35]. In addition, quantification of cl.SNAP25_197_ revealed that BoNT/A activity was dose-dependent, with a maximum positive signal observed in the 30 U/kg subgroup that decreased with attenuated BoNT/A dosages 10 days after the drug injection (Fig. 2c). Furthermore, cl.SNAP25_197_ was detected 10 days following BoNT/A injection and persisted in the lFN for at least 7 weeks following BoNT/A injection at 10 U/kg (Fig. 2d–e).

To further address whether BoNT/A utilized transsynaptic activity to reach the synaptic terminals of second-order projecting neurons, we used an indirect strategy to detect the subcellular spatial localization of SNAP25. SNAP25 was co-immunostained with choline acetyltransferase (ChAT, a marker of cholinergic neurons) and postsynaptic density-95 (PSD95, a dendrite marker representing the post-synaptic membrane). However, SNAP25 surrounded ChAT and PSD95 but did not co-localize with either ChAT or PSD95 (Fig. 2f–g). SNAP25 was thus considered to specifically localize in the plasma membrane of the axons and was acted upon by BoNT/A at this site, as shown through transsynaptic tracing from the cholinergic somas of wFMNs.

**Fig. 3:**
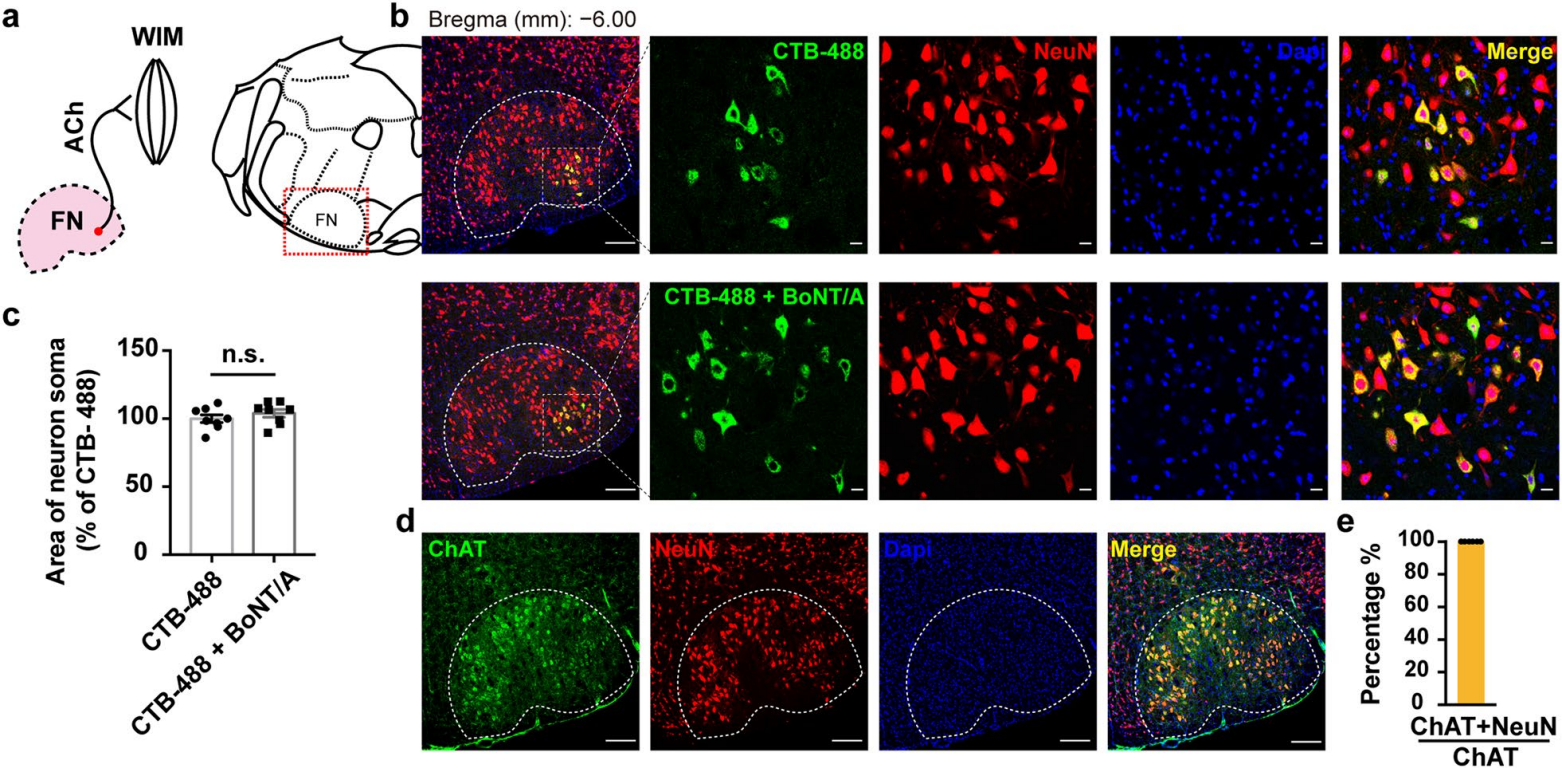
Location and soma size of wFMNs post-BoNT/A WIM injection. **a** Schematic of the pathway of lFN at site of wFMNs projecting to WIM. **b** CTB-488-labelled neurons were all NeuN-positive, locating in the lFN 10 days after single CTB-488 or CTB-488-mixed BoNT/A injected into unilateral WIM. Scale bar, 500 μm (left one); 50 μm (right four). **c** Comparison of the soma size of effected neurons on lFN between the groups of single CTB-488 and CTB-488-mixed BoNT/A injection. *n* = 3 mice per group, unpaired two-tailed student’s *t*-test, *t* = 0.9621, *df* = 14, *P* = 0.3523. The neuron soma area in each group was normalized to the group of mice injected with single CTB-488. **d** Representative co-immunostaining image of ChAT and NueN. Scale bar, 500 μm. **e** All NeuN-positive cells expressing ChAT in the FN (*n* = 6 brain sections from 3 mice). n.s., non-significance; ACh, acetyl cholinergic pathway

Next, we used the monosynaptic retrograde tracer CTB-488 to label the wFMNs by injecting CTB-488 alone or in combination with BoNT/A (CTB-488-mixed BoNT/A [36]) into the unilateral WIM under condition of trigeminal ganglion infraorbital nerve transection. After 10 days, CTB-488 signals were detected only in the ipsilateral lFN of the injection side, but not in the trigeminal sensory nuclear complex (Fig. 3b, and Additional file 2: Fig. S4a). Co-immunostaining for NeuN (a neuronal marker) together with CTB-488 and ChAT confirmed that the wFMNs were all cholinergic (Fig. 3b and d–e). Measurement of the NeuN positive cell size of co-labelled neurons between the CTB-488-mixed BoNT/A and CTB-488-alone groups revealed no change in wFMNs soma size following retrograde BoNT/A (Fig. 3c).

To identify the terminal types of afferent axons projecting to wFMNs, we focused on three distinct types of synapses expressing acetylcholine vesicular transporter (vAChT), serotonin transporter (SerT), or vesicular glutamate transporter 2 (vGluT2), which had been verified to innervate the lFN [37]. Co-immunostaining of these specific synaptic markers in brain sections from CTB-488-mixed BoNT/A-treated mice revealed that these types of fibres all sent projections to wFMNs (Additional file 2: Fig. S4b–e), illustrating large excitatory neurons of the premotor nucleus.

### Anatomical neural connectivity of the ipsilateral vlPAG–wFMNs–WIM

**Fig. 4:**
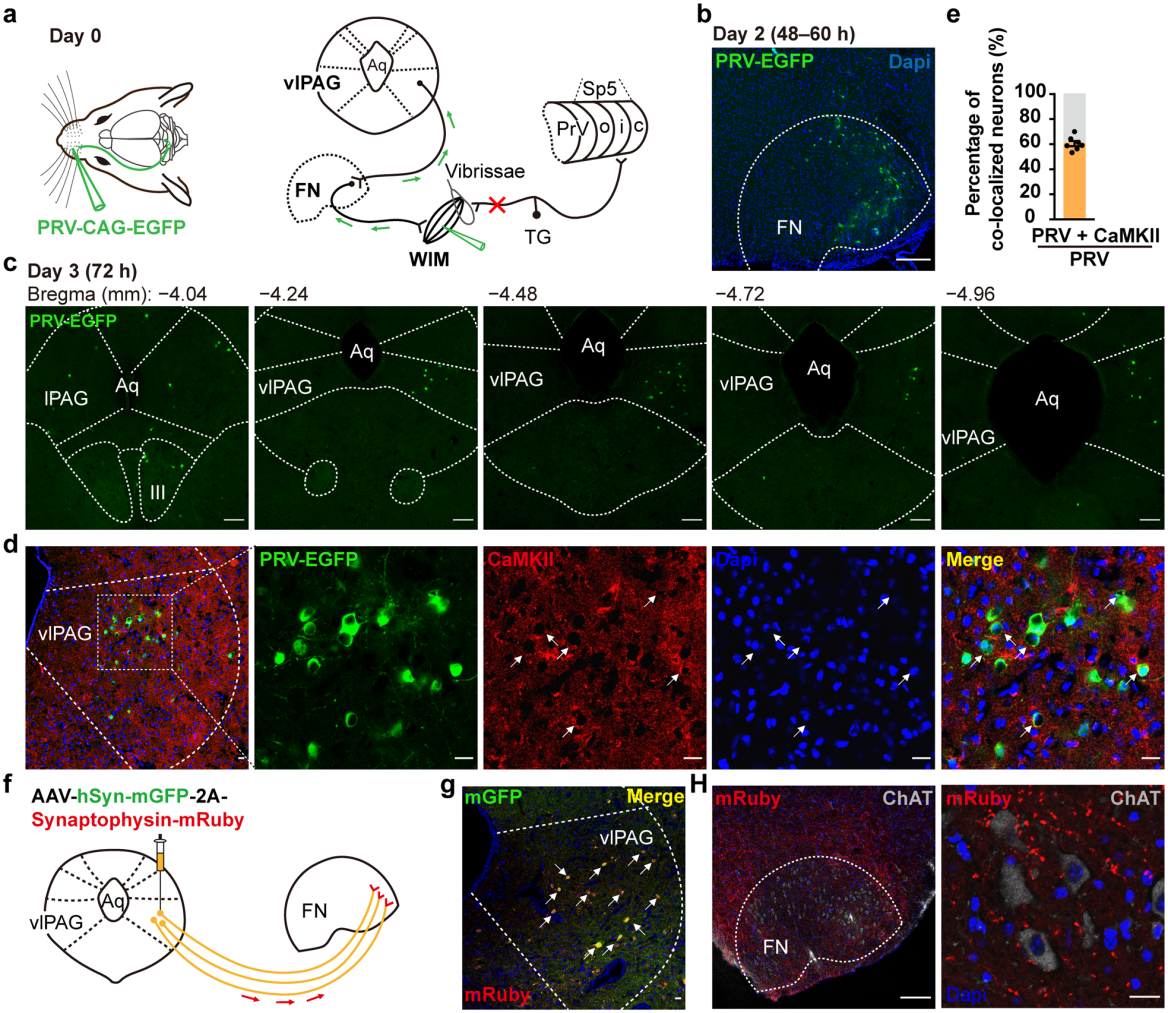
The vlPAG is an anatomical upstream of WIM. **a** Retrograde tracing strategy of tracing the upstream of WIM through the transsynaptic retrograde PRV-EGFP vector injection into the unilateral WIM of mice with transected infraorbital branch of the maxillary nerve of the trigeminal ganglion. **b** The primary order of WIM, which was labelled by PRV-EGFP green signals was located in the lateral subnucleus of FN (lFN) 48–60 h after the virus injection. Scale bar, 500 μm. **c** Representative images of one side of the rostral to caudal vlPAG showed PRV-EGFP-labelled neurons 72 h later of PRV-EGFP infections (*n* = 3 mice per group). Scale bar, 500 μm. **d** Representative image of PRV-EGFP infectious neurons in the unilateral vlPAG that were co-labelled with CaMKII-positive neurons. Scale bar, 200 μm (left one); 50 μm (right four). **e** Percentage of total PRV-EGFP infectious neurons expressing CaMKII in the ipsilateral vlPAG of injection side (*n* = 7 brain sections from 3 mice). **f** Scheme of verifying vlPAG descending output fibres onto the ipsilateral wFMNs upon a monosynaptic anterograde virus vector infused into the unilateral vlPAG. **g** Typical image of unilateral vlPAG with starter cells labelled with AAV-hSyn-mGFP-2A-Synaptophysin-mRuby (yellow). Scale bar, 200 μm. **h** Representative image of Synaptophysin-mRuby-labelled synaptic fibres and terminals innervating ChAT positive neurons in the ipsilateral lFN. The right image was the magnified view of the left one. Scale bar, 500 μm (left); 50 μm (right). Aq, aqueduct; TG, trigeminal ganglion; lPAG, lateral PAG

To investigate the circuitry upstream of wFMNs, which might be potential nucleus BoNT/A retrograde and transsynaptic entrances, we applied a retrograde polytranssynaptic pseudorabies virus (PRV) tracer. EGFP-conjugated PRV (PRV-EGFP) was injected into the unilateral WIM of animals with a transected infraorbital nerve of the trigeminal ganglion to rule out interference from afferent sensory regions (Fig. 4a). The mice were sacrificed every 12 h from 24 to 96 h post-PRV infection, and whole brain sections were evaluated to determine the optimal time gradient for exploring the projection order. Soma mapping revealed that only the unilateral lFN was labelled, starting at 48–60 h following PRV-EGFP injection (Fig. 4b and Additional file 2: Fig. S5a), which were the primary nucleus of WIM. At 72 h, PRV-EGFP-positive neurons were distributed among several midbrain regions (considered the secondary nucleus of WIM), including the vlPAG (Fig. 4c), parvicellular reticular nucleus, lateral paragigantocellular nucleus, and parabrachial nucleus (Additional file 2: Fig. S5b), consistent with previous reports [23, 24]. Furthermore, at 96 h post-PRV-EGFP injection, higher-order regions, including the midbrain, such as the dorsal raphe nucleus, and forebrain, such as the amygdala, thalamus, and motor cortex, were labelled by PRV-EGFP (Additional file 2: Fig. S6). As depression has been reported to be implicated in dysfunction of the midbrain’s vlPAG, we thus targeted the wFMNs-projecting vlPAG as a potential region that might modulate the antidepressant function of retrograde BoNT/A following facial injection.

To further ascertain the anatomical synaptic connectivity of vlPAG input to wFMNs, we visualized the vlPAG terminals in the lFN through monosynaptic antegrade viral expression of membrane-tethered GFP and the presynaptic marker synaptophysin-mRuby under control of the human synapsin (hSyn) promoter (Fig. 4f). At 3 weeks following unilateral infusion of the vlPAG, co-immunostaining of the brain sections encompassing the lFN with ChAT revealed that mRuby-labelled fibres and terminals could be observed in the cholinergic neurons of the lFN (Fig. 4g, h). Both antegrade and retrograde neural tracing confirmed the synaptic connection of the vlPAG afferent to the ipsilateral wFMNs.

Next, to identify the neuron types in the vlPAG that send afferent synapses to the wFMNs, the brain sections including the vlPAG from animals sacrificed at 72 h following PRV-EGFP infection were immunolabelled using excitatory (Ca^2+^/calmodulin-dependent protein kinase type II (CaMKII)), inhibitory (glutamate decarboxylase 1 (GAD67)), dopaminergic (tyrosine hydroxylase (TH)), and serotonergic (tryptophan hydroxylase 2 (TpH2)) neuronal markers (Fig. 4d and Additional file 2: Fig. S7). Approximately 60% of wFMNs-projecting vlPAG neurons were CaMKII-positive (Fig. 4e).

### BoNT/A inhibits the overexcitation of excitatory neurons in the vlPAG induced by CRS

To evaluate whether the activity of wFMNs-projecting vlPAG neurons is altered following CRS or BoNT/A application, we examined the expression of c-Fos, an immediate early protein used to detect neural activity, in the vlPAG among the subgroups of naïve and CRS mice with saline or BoNT/A (10 U/kg) administration 3 weeks prior to the end of CRS. The cl.SNAP25_197_ was extremely significantly higher in the subgroup of 10 U/kg than the 3 U/kg (Fig. 2b, c). Even though the cl.SNAP25_197_-positive signals in the 30 U/kg subgroup were higher than the subgroup of 10 U/kg BoNT/A (Fig. 2b, c), this dosage to cleave the SNAP25 was close to the ceiling effect, and also much higher than the maximum clinical dosage. The 10 U/kg is considered as one of the therapeutic doses in mice, thus the dosage of 10 ug/kg is used in the following research.

**Fig. 5:**
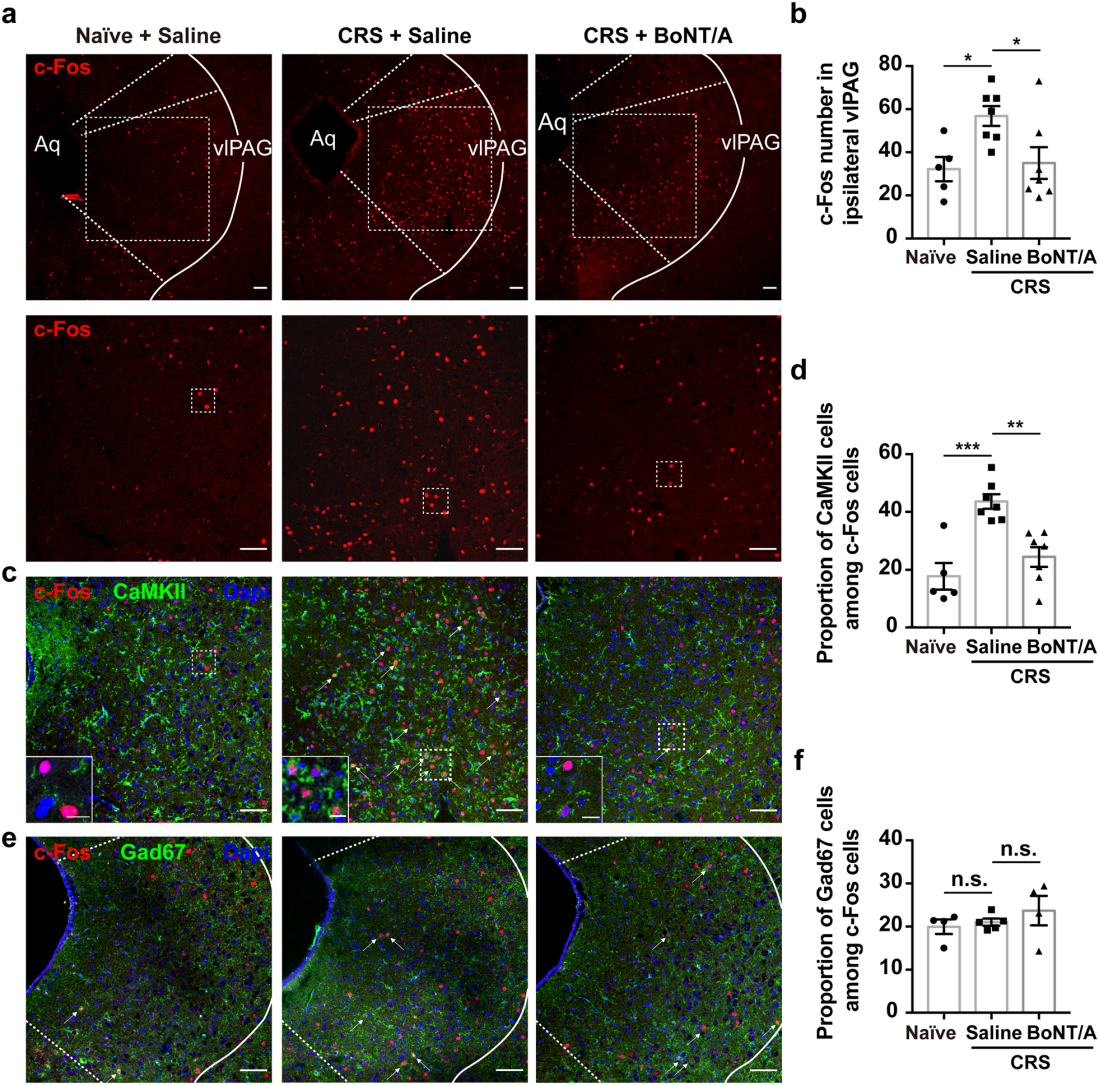
Facial pre-injection of BoNT/A inhibits the overexcitation of CaMKII-positive neurons in the vlPAG of CRS mice. **a** Representative images of the c-Fos expression in the ipsilateral vlPAG to WIM injection side among subgroups of naïve and CRS-induced depression mice 3 weeks post-saline or BoNT/A (10 U/kg) pre-injection. Bottom, magnified view of images indicated in the white rectangle of upper images. Scale bar, 500 μm. **b** Quantificational analysis of c-Fos numbers among subgroups of naïve mice, saline and BoNT/A pre-injected CRS mice. *n* = 3 mice per subgroup, One-way ANOVA test and Dunnett’s multiple comparisons test comparing each subgroup with the subgroup of CRS mice pre-treated with saline, *F*_(2,__16)_ = 4.967, *P* = 0.0210. The *P*-value of Dunnett’s multiple comparisons: Naïve + Saline vs. CRS + Saline: *P* = 0.0274, CRS + Saline vs. CRS + BoNT/A: *P* = 0.0321. **c** c-Fos co-labelled with CaMKII-positive neurons. Bottom left, magnified view of the image indicated in white rectangle. Scale bar, 200 μm; 50 μm (left bottom). **d** Proportion of c-Fos and CaMKII double-labelled neurons in c-Fos-labelled neurons. *n* = 3 mice per group, Dunnett’s multiple comparisons test comparing each subgroup with the subgroup of CRS mice who were pre-treated with saline, *F*_(2,__16)_ = 15.39, *P* = 0.0002. The *P*-value of Dunnett’s multiple comparisons: Naïve + Saline vs. CRS + Saline: *P* = 0.0002, CRS + Saline vs. CRS + BoNT/A: *P* = 0.0013. **e** Representative images of c-Fos co-labelled with GAD67. Scale bar, 500 μm. **f** Proportion of c-Fos and Gad67 double-labelled neurons in c-Fos-labelled neurons. *n* = 3 mice per group, Dunnett’s multiple comparisons test comparing each subgroup with the subgroup of CRS mice pre-treated with saline, *F*_(2,__10)_ = 0.7974, *P* = 0.4772. The *P*-value of Dunnett’s multiple comparisons: Naïve + Saline vs. CRS + Saline: *P* = 0.9102, CRS + Saline vs. CRS + BoNT/A: *P* = 0.5721. *P < 0.05, **P < 0.01, ***P < 0.001, n.s., non-significance. Note: Naïve subgroup: naïve mice treated with saline

We found that whereas c-Fos expression was significantly increased in the vlPAG of CRS mice that received saline, BoNT/A whisker pad injection significantly reduced the number of c-Fos-positive neurons (Fig. 5a, b). Moreover, immunofluorescence revealed that nearly 50% of the activated c-Fos-positive neurons in CRS mice were colocalized with CaMKII-positive neurons (Fig. 5c, d), whereas almost 20% were Gad67 positive (Fig. 5e, f). The number of c-Fos and CaMKII double-positive neurons was strikingly decreased in the vlPAG of BoNT/A-treated CRS mice (Fig. 5c, d).

### Inhibition of vlPAG–wFMNs excitatory neurons of CRS mice mimics facial BoNT/A antidepressant effects

**Fig. 6:**
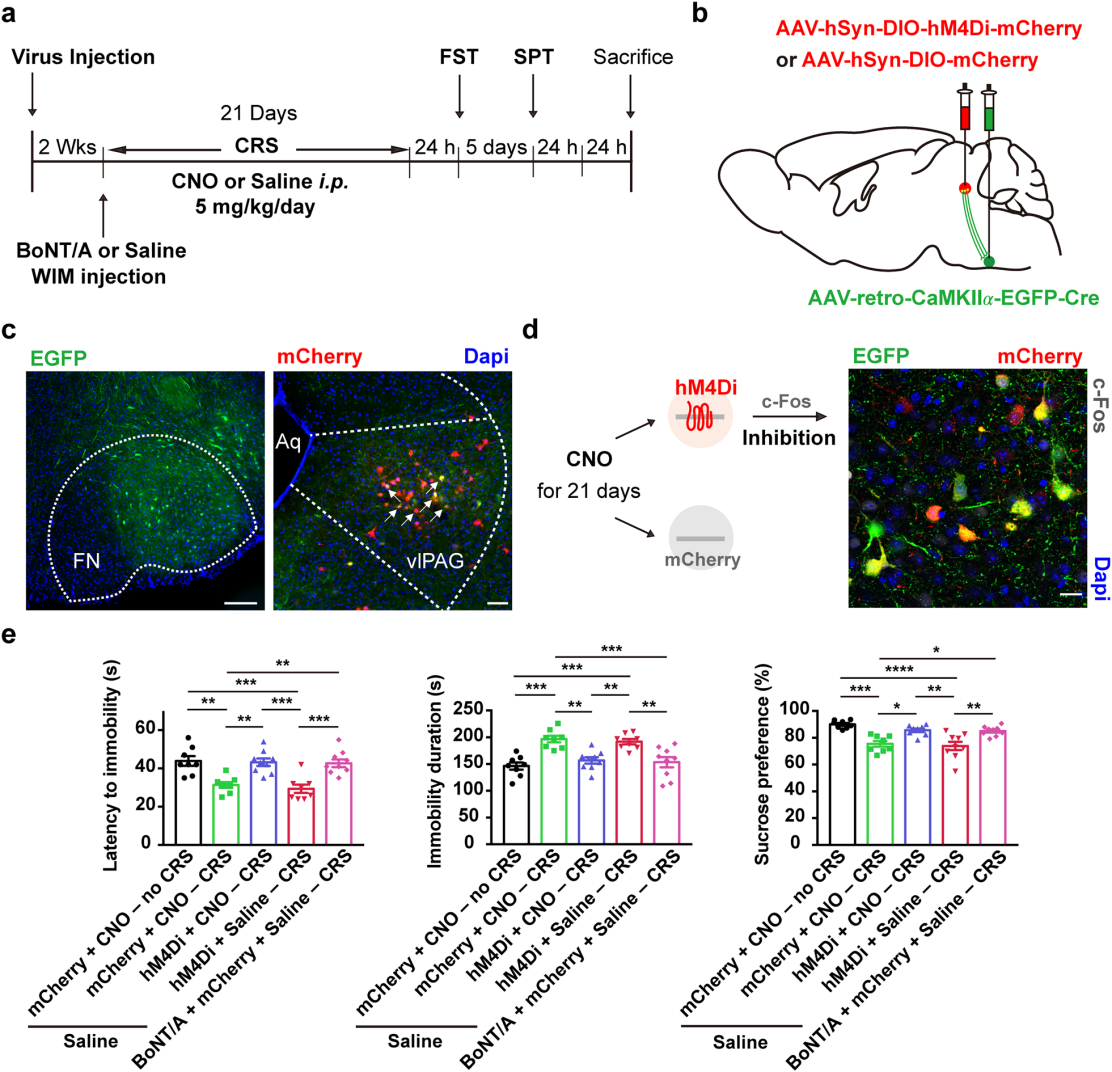
Inhibition of wFMNs-projecting vlPAG excitatory neurons improves the depressive-like behavior induced by CRS. **a** Schematic of experimental design. **b** Scheme for specific infection of wFMNs-projecting vlPAG excitatory neurons with hM4Di or mCherry by stereotaxic infusion of rAAV-retro-CaMKIIα-EGFP-Cre into the unilateral lFN at the site of wFMNs and AAV-DIO-hM4Di-mCherry or AAV-DIO-mCherry into the ipsilateral vlPAG. **c** Representative images of lFN (left) and vlPAG (right) 2 weeks after virus injection. Scale bar, 500 μm (left); 200 μm (right). **d** Representative image of the vlPAG indicated that few c-Fos expression was co-labelled with EGFP and mCherry double-positive neurons after 3-week continuous *i.p.* injection of CNO. **e** Behavioral despair in the FST (left and middle) and anhedonia in the SPT (right) among experimental groups. All mice injected by a rAAV-retro-CaMKIIα -EGFP-Cre vector into the unilateral lFN at the site of wFMNs. Saline + mCherry + CNO − no CRS, mice that received WIM pre-injection of saline, vlPAG injection of AAV-DIO-mCherry, and *i.p.* injection of CNO without exposure to CRS. Saline + mCherry + CNO − CRS, mice that received WIM pre-injection of saline, vlPAG injection of AAV-DIO-mCherry, and *i.p.* injection of CNO with exposure to CRS. Saline + hM4Di + CNO − CRS, mice that received WIM pre-injection of saline, vlPAG injection of AAV-DIO-hM4Di-mCherry, and *i.p.* injection of CNO with exposure to CRS. Saline + hM4Di + Saline − CRS, mice that received WIM pre-injection of saline, vlPAG injection of AAV-DIO-hM4Di-mCherry, and *i.p.* injection of saline with exposure to CRS. BoNT/A + mCherry + Saline − CRS, mice that received WIM pre-injection of BoNT/A, vlPAG injection of AAV-DIO-mCherry, and *i.p.* injection of saline with exposure to CRS. n = 8 animals from the group of Saline + mCherry + CNO − no CRS, Saline + mCherry + CNO − CRS and Saline + hM4Di + Saline − CRS, n = 9 animals from the group of Saline + hM4Di + CNO − CRS and BoNT/A + mCherry + Saline − CRS. One-way ANOVA followed by Bonferroni’s multiple comparisons test, *F*_(4,__37)_ = 11.63, *P* < 0.0001 for latency to immobility in the FST; *F*_(4,__37)_ = 10.92, *P* < 0.0001 for immobility duration in the FST; *F*_(4,__37)_ = 12.22, *P* < 0.0001 for sucrose preference in the SPT. The *P*-value of Bonferroni’s multiple comparisons: Saline + mCherry + CNO − no CRS *vs.* Saline + mCherry + CNO − CRS: *P* = 0.0017, Saline + mCherry + CNO − no CRS *vs.* Saline + hM4Di + Saline–CRS: *P* = 0.0002, Saline + mCherry + CNO − CRS *vs.* Saline + hM4Di + CNO − CRS: *P* = 0.0023, Saline + mCherry + CNO − CRS *vs.* BoNT/A + mCherry + Saline − CRS: *P* = 0.0040, Saline + hM4Di + CNO − CRS *vs.* Saline + hM4Di + Saline − CRS: *P* = 0.0003, Saline + hM4Di + Saline − CRS *vs.* BoNT/A + mCherry + Saline − CRS: *P* = 0.0005 of latency of immobility; Saline + mCherry + CNO − no CRS *vs.* Saline + mCherry + CNO − CRS: *P* = 0.0001, Saline + mCherry + CNO − no CRS *vs.* Saline + hM4Di + Saline − CRS: *P* = 0.0006, Saline + mCherry + CNO − CRS *vs.* Saline + hM4Di + CNO − CRS: *P* = 0.0022, Saline + mCherry + CNO − CRS *vs.* BoNT/A + mCherry + Saline − CRS: *P* = 0.0008, Saline + hM4Di + CNO − CRS *vs.* Saline + hM4Di + Saline–CRS: *P* = 0.0092, Saline + hM4Di + Saline − CRS *vs.* BoNT/A + mCherry + Saline–CRS: *P* = 0.0036 of total time of immobility; Saline + mCherry + CNO − no CRS *vs.* Saline + mCherry + CNO − CRS: *P* = 0.0001, Saline + mCherry + CNO − no CRS *vs.* Saline + hM4Di + Saline − CRS: *P* < 0.0001, Saline + mCherry + CNO − CRS *vs.* Saline + hM4Di + CNO − CRS: *P* = 0.0123, Saline + mCherry + CNO − CRS *vs.* BoNT/A + mCherry + Saline − CRS: *P* = 0.0160, Saline + hM4Di + CNO − CRS *vs.* Saline + hM4Di + Saline − CRS:* P* = 0.0017, Saline + hM4Di + Saline − CRS *vs.* BoNT/A + mCherry + Saline − CRS: *P* = 0.0022 of sucrose preference. *P < 0.05, **P < 0.01, ***P < 0.001, ****P < 0.0001

To verify that the vlPAG–wFMNs pathway is involved in the antidepressant effects of BoNT/A facial injection, we used region-specific chemogenetic manipulation to control the wFMNs-projecting excitatory neurons in the vlPAG. Firstly, a monosynaptic retrograde virus anchored with Cre for expressing EGFP under the control of the CaMKIIα promotor (rAAV-retro-CaMKIIα-EGFP-Cre) was unilaterally infused into the lFN at the site of wFMNs. Then, wFMNs-projecting vlPAG excitatory neurons were infected with another Cre-dependent virus encoding a neural inhibitor hM4Di and mCherry expression (AAV-hSyn-DIO-hM4Di-mCherry) infused into the ipsilateral vlPAG (Fig. 6b, c). A virus expressing only mCherry (AAV-hSyn-DIO-mCherry) was used as a control. After 2 weeks of incubation to allow for virus expression, mice were subjected to 3-week CRS accompanied by daily *i.p.* injection of CNO or saline. Repeated inhibition of wFMNs-projecting excitatory neurons in the vlPAG over 21 continuous days, represented by few expressions of c-Fos with EGFP and mCherry double-positive neurons (Fig. 6d and Additional file 2: Fig. S8), was sufficient to reverse the CRS-induced depressive-like behavior without locomotion ability altered (Fig. 6e and Additional file 2: Fig. S8), which mimicked the antidepressant function of facial injected BoNT/A.

### wFMNs-projecting vlPAG excitatory neuron activation neutralizes the BoNT/A antidepressant function

**Fig. 7:**
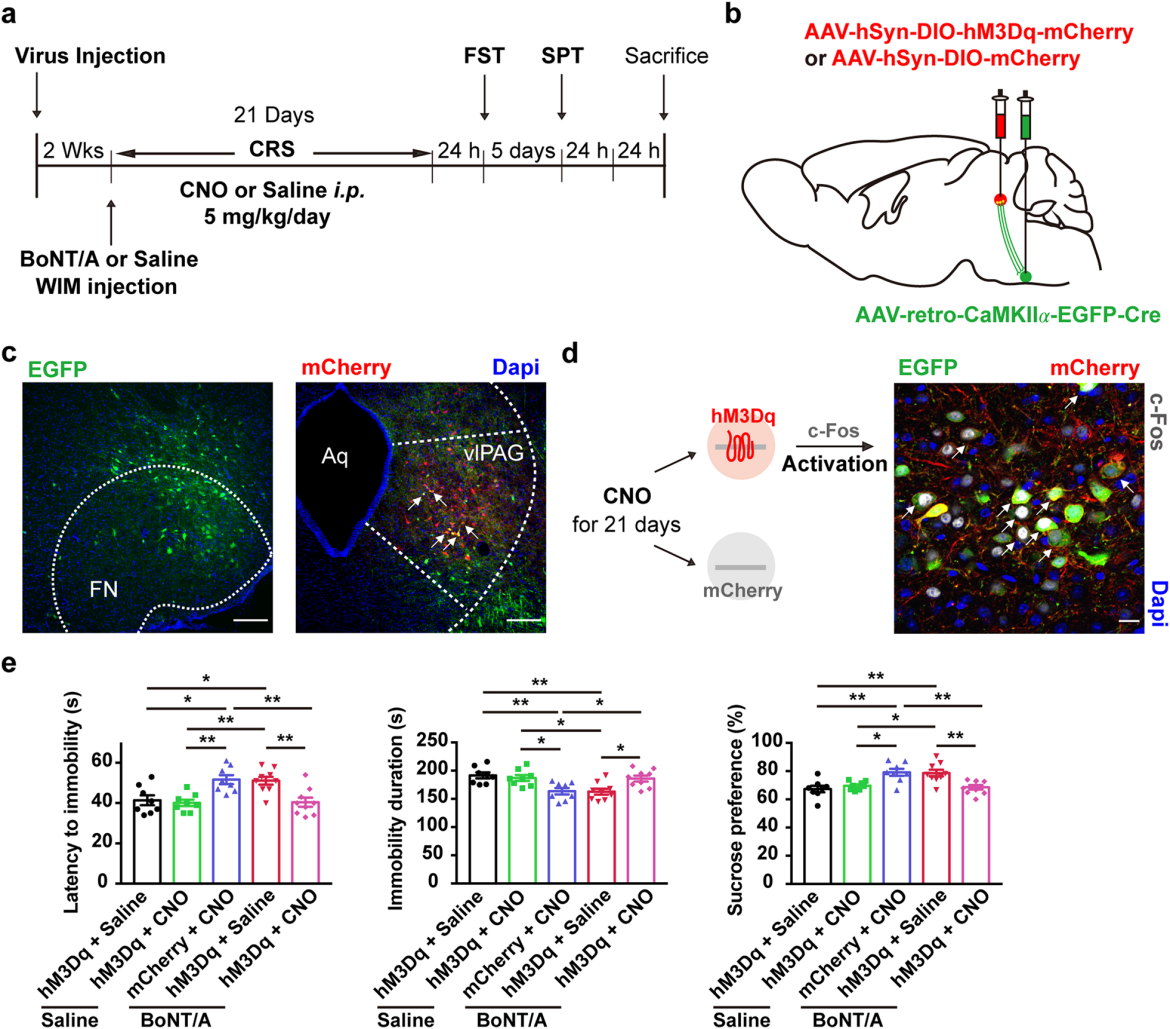
Excitation of wFMNs-projecting vlPAG excitatory neurons attenuates the antidepressant-like behavior of facial BoNT/A. **a** Schematic of experimental design. **b** Scheme for specific infection of wFMNs-projecting vlPAG excitatory neurons with hM3Dq or mCherry by stereotaxic infusion of rAAV-retro-CaMKIIα-EGFP-Cre into the unilateral lFN at the site of wFMNs and AAV-DIO-hM3Dq-mCherry or AAV-DIO-mCherry into the ipsilateral vlPAG. **c** Representative images of lFN (left) and vlPAG (right) 2 weeks after virus injection. Scale bar, 500 μm. **d** Representative image of the vlPAG indicated that *i.p.* injection of CNO evoked c-Fos expressions in neurons expressing hM3Dq. Scale bar, 100 μm. **e** Behavioral despair in the FST (left and middle) and anhedonia in the SPT (right) among experimental groups. All mice injected by a rAAV-retro-CaMKIIα-EGFP-Cre vector into unilateral lFN and exposure to CRS. Saline + hM3Dq + Saline, CRS mice that received WIM pre-injection of saline, vlPAG injection of AAV-DIO-hM3Dq-mCherry, and *i.p.* injection of saline. Saline + hM3Dq + CNO, CRS mice that received WIM pre-injection of saline, vlPAG injection of AAV-DIO-hM3Dq-mCherry, and *i.p.* injection of CNO. BoNT/A + mCherry + CNO, CRS mice that received WIM pre-injection of BoNT/A, vlPAG injection of AAV-DIO-mCherry, and *i.p.* injection of CNO. BoNT/A + hM3Dq + Saline, CRS mice that received WIM pre-injection of BoNT/A, vlPAG injection of AAV-DIO-hM3Dq-mCherry, and *i.p.* injection of saline. BoNT/A + hM3Dq + CNO, CRS mice that received WIM pre-injection of BoNT/A, vlPAG injection of AAV-DIO-hM3Dq-mCherry, and *i.p.* injection of CNO. n = 8 animals from the group of Saline + hM3Dq + Saline, Saline + hM3Dq + CNO and BoNT/A + mCherry + CNO, n = 9 animals from the group of BoNT/A + hM3Dq + Saline and BoNT/A + hM3Dq + CNO. One-way ANOVA followed by Bonferroni’s multiple comparisons test, *F*_(4,__37)_ = 7.705, *P* = 0.0001 for latency to immobility in the FST; *F*_(4,__37)_ = 6.778, *P* = 0.0003 for immobility duration in the FST; *F*_(4,__37)_ = 8.012, *P* < 0.0001 for sucrose preference in the SPT. The *P*-value of Bonferroni’s multiple comparisons: Saline + hM3Dq + Saline *vs.* BoNT/A + mCherry + CNO: *P* = 0.0199, Saline + hM3Dq + Saline *vs.* BoNT/A + hM3Dq + Saline: *P* = 0.0244, Saline + hM3Dq + CNO *vs.* BoNT/A + mCherry + CNO: *P* = 0.0063, Saline + hM3Dq + CNO *vs.* BoNT/A + hM3Dq + Saline: *P* = 0.0076, BoNT/A + mCherry + CNO *vs.* BoNT/A + hM3Dq + CNO: *P* = 0.0063, BoNT/A + hM3Dq + Saline *vs.* BoNT/A + hM3Dq + CNO: *P* = 0.0075 of latency of immobility; Saline + hM3Dq + Saline *vs.* BoNT/A + mCherry + CNO: *P* = 0.0084, Saline + hM3Dq + Saline *vs.* BoNT/A + hM3Dq + Saline: *P* = 0.0049, Saline + hM3Dq + CNO *vs.* BoNT/A + mCherry + CNO: *P* = 0.0421, Saline + hM3Dq + CNO *vs.* BoNT/A + hM3Dq + Saline: *P* = 0.0265, BoNT/A + mCherry + CNO *vs.* BoNT/A + hM3Dq + CNO: *P* = 0.0458, BoNT/A + hM3Dq + Saline *vs.* BoNT/A + hM3Dq + CNO: *P* = 0.0284 of total time of immobility; Saline + hM3Dq + Saline *vs.* BoNT/A + mCherry + CNO: *P* = 0.0028, Saline + hM3Dq + Saline *vs.* BoNT/A + hM3Dq + Saline: *P* = 0.0034, Saline + hM3Dq + CNO *vs.* BoNT/A + mCherry + CNO: *P* = 0.0258, Saline + hM3Dq + CNO *vs.* BoNT/A + hM3Dq + Saline: *P* = 0.0323, BoNT/A + mCherry + CNO *vs.* BoNT/A + hM3Dq + CNO: *P* = 0.0068, BoNT/A + hM3Dq + Saline *vs.* BoNT/A + hM3Dq + CNO: *P* = 0.0083 of sucrose preference. *P < 0.05, **P < 0.01

Next, we infused a Cre-dependent virus expressing neural excitator hM3Dq and mCherry (AAV-hSyn-DIO-hM3Dq-mCherry) unilaterally into the vlPAG concurrent with rAAV-retro-CaMKIIα-EGFP-Cre infusion into the ipsilateral lFN at site of wFMNs (Fig. 7b). Two weeks following virus injection, mice were administered CNO intraperitoneally once daily for three weeks to activate the wFMNs-projecting CaMKIIα-positive neurons in the vlPAG and a WIM injection of 10 U/kg BoNT/A or saline at one day prior to the first day of CNO administration (Fig. 7a). However, daily activation of vlPAG–wFMNs excitatory neurons were represented by triple-positive c-Fos, EGFP, and mCherry staining (Fig. 7d and Additional file 2: Fig. S9), diminished BoNT/A-promoted behavioral anhedonia and despair as reflected in the FST and SPT performance of CRS mice (Fig. 7e). Of note, chemogenetic daily activation of wFMNs-projecting vlPAG excitatory neurons decreased total travel distance and time in the center zone in the OFT compared to the behaviors of non-CNO-exposed CRS mice; nevertheless, BoNT/A treatment reversed the reduced travel distance rather than the time in the center zone in the OFT (Additional file 2: Fig. S9).

## Discussion

BoNT/A facial procerus and corrugator injection improves depressive symptoms in patients with MDD [10–13]; however, the underlying neuronal loop based on retrograde BoNT/A action remains largely obscure. Here, using a CRS depression mouse model, we found that BoNT/A undergoes retrograde cell-to-cell transport from facial injection loci to the second-order neuron wFMNs-projecting synaptic boutons could directly modulate depression by inhibiting the excitatory neurons in the vlPAG with output to the wFMNs.

Pre-injection of high dose of 30 U/kg BoNT/A at three time points, while mice in the 10 U/kg subgroups pre-injected BoNT/A 3 weeks and 3 days, and 3 U/kg subgroup pre-administered BoNT/A 3 weeks prior to the end of restraint robustly exhibited ameliorated depressive-like behavior. These results indicated that under a certain dosage, the reaction and duration of the BoNT/A effect on depressive behavior is related to the drug dosage. Specifically, higher dosage of BoNT/A induced faster effects and lasts for longer effectiveness, depending on the endochylema concentration and mouse metabolism. Interestingly, the peripheral action represented by the WIM paralysis could be observed at 1 day after the BoNT/A injection of three dosages, but in the 3 days after the 3 U/kg of BoNT/A injection, CRS mice didn’t exhibit the antidepressant behavior. The 3-day might be too short for a low dosage (3 U/kg) of BoNT/A to take retrograde transport or take central effects on the depressive-like behaviors of mice. Further experiment design including a broader time window is needed to explore the specific efficiency time of the BoNT/A.

The antidepressant effect of BoNT/A injection in the glabellar region is a result of both indirect and direct roles [10–13, 38, 39]. It was hypothesized that the reciprocal facial rejuvenation and proactive emotional expressions following BoNT/A relieve the corrugator and yield positive social feedback [14]. In addition, according to the hypothesis of facial feedback involving embodying emotion, as it is impossible to make an unpleasant facial expression when the corrugator is paralyzed, the evolvement and adaption of negative feelings is therefore reduced [15]; meanwhile, some central emotional feedback might be influenced by the altered afferent signals from flaccid facial muscles upon BoNT/A injection [16]. However, results obtained from rodents are not generalizable to human feelings and expression. Moreover, the causality between facial expressions and emotion processing remains controversial. In our study, unilateral facial BoNT/A injection led to asymmetrical whisker apraxia. Asymmetric paralysis leads to the restriction of vibrissae sweeping, which can negatively influence the emotions of mice [21, 40]. Whisking, which depends on musculation and vibrissae sweeps, is essential for spatial navigation, discrimination, exploration, and even balance in rodents [41–43]. For example, malfunction of whisker-dependent tactile perception can cause negative social behavior and diffuse emotion processing in mice [20]; whisker deprivation in adult mice could decrease the time spent in the central region of the OFT but not the total distance travelled, and negatively alter the short-term learning ability in a novel object recognition test, without changing the immobility time in the FST [21]. In contrast, we previously observed that unilateral facial BoNT/A application enhanced the learning ability of naïve mice in a Morris water maze test [19]. Notably, together with Li et al. [17], our current results based on unilateral whisker activity deprivation by BoNT/A that CRS mice exhibit antidepressant-like behavior as reflected by decreased immobility duration and increased immobility latency in the FST, without abnormal OFT locomotor activity, and elevated glucose preferences in the SPT were also contradictory to the reported phenomena observed following vibrissae trimming [20, 21, 40], indicating the potential for the central action of BoNT/A.

BoNT/A craniofacial muscular injection is followed by central reorganization or neuronal network remodeling. Reduced functional connectivity of the sensorimotor cortex and right superior frontal gyrus was found in patients with torsion cervical dystonia treated with a single BoNT/A injection [44]. Decreased activation of the basal ganglia–thalamic pathway was observed following BoNT/A injection in the forehead and periorbital region of patients with Meige’s syndrome [6]. However, the central effects, including decreased activation of some brain regions following BoNT/A peripheral administration, cannot be simply explained by a reduction in spindle afferents to the CNS induced through denervation of the neuromuscular junction whereas with few proprioceptors in facial muscles. The specific mechanism is complex and requires further study. Several studies have revealed that BoNT/A undergoes retrograde transport and transcytosis to exert distant effects on central areas upstream of the injection loci. In vitro studies using cultured hippocampal and cortical neurons revealed that BoNT/A undergoes retrograde transport along axons, followed by cell-to-cell transfer into the synaptic terminals of upstream neurons, and SNAP25 cleavage [8, 45]. In rodents, activity blockade following cl.SNAP25_197_ occurred in the contralateral hemisphere after BoNT/A infusion into one side of the hippocampus [7]. Truncated-SNAP25 was detected in retina neurons two synapses away from the injection site 3 days after BoNT/A was injected into the optic tectum of rats [46, 47], and we previously detected cl.SNAP25_197_ in the lumbosacral spinal cord following BoNT/A gastrocnemius injection [48].

Our current finding that BoNT/A-cl.SNAP25_197_ was detectable only in one side of the lFN, a subnucleus encompassing upstream synaptic terminals without interneurons, but not in the trigeminal sensory nuclear complex, adjacent regions, or premotor nucleus, parallels the concept of BoNT/A retrograde transcytosis via neural connection rather than systemic diffusion. Cholinergic motor neurons innervating the WIM are located in the slightly ventral lFN whereas the retractor extrinsic muscle neurons are located on the dorsolateral site [24, 49]. cl.SNAP25_197_ was limited to the ventral lFN following 3 or 10 U/kg BoNT/A administration, whereas a few positive signals were detected in the dorsolateral FN except in the surrounding area upon 30 U/kg BoNT/A application in the WIM. This may have occurred because an overdose of the toxin might diffuse to the retractor extrinsic muscles in a finite radius around the injection point; nevertheless, these findings rule out the possibility of BoNT/A systemic spread beyond the injection loci, although it held biological activity in the CNS. Furthermore, the antidepressant behavior (lasting for at least 6 weeks) of BoNT/A-treated CRS mice in our study exceeded the period of injected muscle paralysis (2–3 weeks). This supports the model in which both distant and direct central effects of BoNT/A regulate emotion via axonal retrograde and transsynaptic transport into wFMNs-projecting synaptic terminals, altering the function of second-order neurons. To achieve a more objective view of BoNT/A retrograde movement and incriminate higher-order brain regions that would be directly affected by peripherally injected BoNT/A, we are currently attempting to construct a tag-conjugated BoNT/A with fully retained functionality to facilitate further research regarding its antidepressant action.

Notably, the follicle-sinus and cutaneous skin of the whisker pad are also innervated by the infraorbital nerve of the trigeminal ganglion, which is composed of pseudounipolar neurons that transport afferent facial sensory signals to the trigeminal sensory nuclear complex [35, 50]. BoNT/A exhibits higher binding affinity to the presynaptic plasma membrane of the skeletal, autonomic cholinergic, and then other excitatory terminals than to inhibitory terminals [9, 51]. BoNT/A could also bind to sensory nerve endings to inhibit the release of neuropeptides in the periphery [52]. However, whether it plays enzymatic roles in the sensory center, directly reducing central sensitization, remains to be established. In this study, we did not detect BoNT/A-cl.SNAP25_197_ in the Pr5 or Sp5, although the nucleus received sensory axonal terminals from the trigeminal ganglion following deep WIM BoNT/A injection, demonstrating that BoNT/A failed to bind to or be transported via trigeminal sensory neurons. On the other hand, deep muscle administration might preferably confine BoNT/A to allow effective binding and retro-traffic in the efferent cholinergic terminals, which occupy the vast majority of the synapses innervating the whisker muscles. In addition, the majority of c-Fos-positive neurons in the vlPAG of CRS mice inhibited by BoNT/A facial injection were CaMKII-positive, whereas the overlap between GABAergic and c-Fos-positive neurons was little affected. This also indicates that BoNT/A displays an affinity for excitatory neurons in the CNS. Combined with the results of vlPAG–wFMNs excitatory neuron specific manipulation, our findings suggest that BoNT/A influences depressive-like behavior, at least by initially acting on whisker motor neurons, followed by entering the premotor nucleus to influence its function. Whether sensory neurons participate in the regulation of emotions by BoNT/A requires further study.

Complex neural bases and circuits underlie facial emotional processing. fMRI has revealed that exposure to negative images leads to increased amygdala activity albeit decreased ventromedial PFC activity, and stronger right connectivity of the bed nucleus of the stria terminalis and CeA, with concurrent higher EMG activity of the corrugator in humans [53, 54]. Alternatively, paralysis of the corrugator and procerus by BoNT/A attenuates the increased amygdala activity observed when the facial muscles frown at angry facial expressions [55, 56]. The amygdala plays a crucial role in processing emotions when facial muscles undergo emotional expression. BoNT/A relieves the facial muscles, altering their afferent feedback, and may also be retrogradely transported to second-order regions that are innervated by the amygdala, thereby influencing the neural loop related to emotion processing.

Studying neural loops following pharmacotherapy is an essential strategy to uncover the convergence point of pathological behaviors and drug actions. The vlPAG has been implicated in crucial region encoding aversive stimulation-induced maladaptive behaviors involving algesia [25, 27], defensive reactions [22], fear and anxiety [57, 58], and depression [32, 59]. The vlPAG contains glutamatergic, the predominant excitatory neurons, GABAergic, scarce serotoninergic, and dopaminergic neurons [60, 61]. Using antegrade and polyneural retrograde tracing, we found that the vlPAG, which integrates strongly reciprocal connections with the PFC, CeA, and hypothalamus [29], sent descending excitatory projections to the wFMNs. The connectivity of the PAG to wFMNs was also ascertained using existing anatomical observations [23, 24]. Moreover, whisker twitches are activated when the PAG is stimulated [62]. Recent evidence has also supported that the vlPAG is involved in the occurrence and progression of depression. For example, a CeA–vlPAG circuit was found to be responsible for nociception-accompanied depression in CRS mice through enhanced inhibition of vlPAG inhibitory interneuron-projecting GABAergic neurons in the CeA, which thereby disinhibited vlPAG local glutamatergic neurons [27]. In addition to the reported malfunction of glutamatergic transmission in the vlPAG in inflammatory bowel disease-induced depression [31], Tovote et al. showed that the activation of vlPAG glutamatergic neurons in a network of CeA–vlPAG–medullary forelimb premotor nuclei that mapped to freezing behavior did not mediate concomitant analgesia [22]. This observation suggests that specific defensive and algetic responses could be regulated by efferent pathways distinct from the vlPAG.

We observed that CaMKII-positive neuron activation in the vlPAG of CRS rodents, as represented by c-Fos expression, could be diminished by facial BoNT/A administration. Moreover, large PAG iron deposits are negatively associated with the response to facially injected BoNT/A in patients with chronic migraine, suggesting that BoNT/A is not effective if the PAG is damaged [63]. This indicates that the PAG is a crucial region for the efficiency of the central response to facially injected BoNT/A. We, therefore, consider that the vlPAG–wFMNs constitutes a potential circuit involved in emotion processing by retrograde facially injected BoNT/A. Our results reveal that the specific hM4Di-mediated inhibition of vlPAG–wFMNs excitatory neurons alleviates the depressive-like behavior of CRS mice, mimicking the antidepressant action of facial pre-treatment with BoNT/A. Conversely, repeated chemogenetic activation of this subpopulation counteracted the attenuated despair- or anhedonia-like behaviors of CRS mice who received prior BoNT/A WIM injection. Our data indicated that BoNT/A was retrogradely transported through the reversed loop of the vlPAG–wFMNs–WIM to directly affect wFMNs-projecting excitatory neurons in the vlPAG and thereby regulated emotion processing, even though activation of this loop was likely to reduce total distance travelled and center zone duration in the OFT. This suggests that separate activation of this pathway might affect some mood-related behaviors. However, further investigation involving various rodent models is necessary to determine whether additional stress factor-induced depressive symptoms could be rescued by BoNT/A facial injection or are related to similar neural circuitry. Moreover, our study did not investigate functional contributions, such as the synaptic connection strength or neuronal activity of vlPAG–wFMNs effected by BoNT/A; this warrants further study using patch clamp recording, calcium imaging, and optogenetic techniques.

## Conclusions

BoNT/A injected into the WIM inhibited wFMNs-projecting vlPAG excitatory neurons via retrograde and cell-to-cell transport to relieve depressive symptoms in CRS mice. As traditional pharmacological options for MDD produce several side effects and many patients experience drug resistance [4], our findings may provide a reliable theoretical basis supporting an updated approach utilizing BoNT/A facial injection for the effective treatment of MDD with a low possibility of side effects, such as myasthenia and pain at the injection loci, which can also be reversed once the neuron endings sprout.

## Methods

### Animals and ethic statement

Specific pathogen-free C57BL/6J male mice (8 weeks of age, about 25 g in weight) purchased from Hangzhou Ziyuan Laboratory Animal Science and Technology Co., LTD were housed in standard transparent plastic cages (330 × 205 × 180 mm) with four mice per cage under a 12-h:12-h light–dark cycle with *ad libitum* access to food and water. Mice were habituated for 1 week before formal experiments.

### Chronic restraint stress (CRS)

CRS was selected as the mouse model of depression. Mice were subjected to CRS by gently placed into 50-ml conical tubes with holes for air flow 2–3 h per day for 21 consecutive days [27, 34]. Meanwhile, the naïve mice were restricted to the food and water with free moving.

### Drugs and administration

BoNT/A (BOTOX^®^, Allergan Inc., Irvine, USA) was dissolved in sterile saline to the concentration of 1 U/10 μl. Mice were randomly divided into three groups for later measurement at three different time points. Each group was then randomly divided into five subgroups for specific treatment. Mice were anesthetized intraperitoneally (*i.p.*) by 1% pentobarbital sodium, followed by drug injections using Hamilton microsyringes sealed with glass microelectrodes. Three different BoNT/A doses (3, 10 or 30 U/kg) were injected into the central left whisker intrinsic musculature (WIM) lowered to a depth of 1.5 mm. Naïve and one subgroup of CRS mice received sterile saline (5 μl) injections. All mice were returned to their home cages for free moving after administration.

### Behavioral testing

We performed behavioral tests to examine behavioral despair, anhedonia and locomotor ability of mice via the forced swimming test (FST), sucrose preference test (SPT) and open field test (OFT) respectively, as previously described [64]. Mice underwent 5-day handling before the behavior testing.

### Forced swimming test (FST)

Mice were gently placed in the center of an arena (12 cm diameters, 25 cm height) of water (23–25 °C) in a room with normal light and swam for 6 min. Water depth was set to prevent mice from touching the bottom with their tails or hindfoot. A video camera positioned in the side to record mice behavior. The immobility time was recorded during the last 4 min when mice remained floating and motionless with only movements necessary for keeping balance in water. The latency to immobility was defined as mice first gave up escaping. All recording counted by a same experimenter who was blind to the experimental grouping.

### Sucrose preference test (SPT)

Mice were first habituated with two bottles of 1% sucrose for 3 days, followed by two bottles of regular water for one day. And then, the water was deprived for 24 h and then exposed to one bottle with regular water and the other with 1% sucrose for 24 h. The bottle position was switched after 12 h to avoid the position preference. Total consumption of regular water or sucrose was measured and the sucrose preference was defined as the ratio of sucrose consumption of total fluid during 24 h:

$$Sucrose\% = Sucrose consumption / (Sucrose consumption + Regular water consumption) \times 100\%$$

### Open field test (OFT)

Mice were placed in the center of a plastic box (40 × 40 × 30 cm) in a room with dark light for 6 min. Before formal test, mice were habituated for single housed in the room for 30 min. During the session, animal movement was videotaped by a video camera positioned above the arena, and the total distance was analyzed by Any-maze software (Any-maze, Stoelting Inc., UK).

### Tissue section preparation

Mice completed the behavioral tests were sacrificed for immunostaining to detect target molecules. Mice were anesthetized with 1% pentobarbital sodium 24 h after behavioral tests, and then intracardially perfused with phosphate-buffered saline (PBS), followed by 4% paraformaldehyde using a peristaltic pump. The brain was extracted and fixed overnight in paraformaldehyde. For immunohistochemistry (IHC), the tissue was cryoprotected in 75% ethyl alcohol for 24 h, and then diverted to a different alcohol and xylene concentration for gradient dehydration. Then, tissues were cut in 6 μm-thick paraffin sections along the sagittal plane. For immunofluorescence (IF), the brain was diverted into the 30% sucrose for 48 h for dehydration, and then embedded in OCT in – 20 °C then cut into 40 μm-thick frozen sections along the sagittal plane. All frozen brain sections were stored in anti-freezing solution (20% glycol, 30% glycerin, and 50% PBS) at – 20 °C until the next procedure.

### Immunostaining

For IHC, the sections were gradient dewaxed by xylene and alcohol, followed by distilled water rinse. Sections were then washed in PBS and transferred to water-bath heating with citric acid buffer for heat antigen retrieval. Sections were incubated in 0.3% H_2_O_2_ for 30 min at room temperature to quench endogenous peroxidase activity, followed by washing in PBS and incubation in normal goat serum (NGS, SL038, Solarbio) in PBST (PBS Triton Tx-100 0.3%) for 30 min. Brain sections were incubated overnight in 4 °C stained for cleaved SNAP25_197_ (Additional file 1: Table S1). The next day, sections were incubated at room temperature for rewarming for 45 min, followed by washing in PBS and incubation with the second antibody (HRP-conjugated goat anti-mouse/rabbit IgG polymer, GTVisionTM III Detection System/Mo&Rb, GK500710, Gene Tech) at room temperature for 30 min. After washing in PBS, sections were reacted in the DAB-developing agent (GTVisionTM III Detection System/Mo&Rb) for coloration, followed by rinsing with distilled water. The nucleus was stained by hematoxylin (G4070, Solarbio), followed by distilled water rinse. All sections were dehydrated using graded ethanol, vitrified by dimethylbenzene, and mounted on gelatin-coated slides to dry. Image acquisition was performed with an Olympus optical microscope using 10× and 20× objective lens.

For IF, sections were incubated in 5% NGS or donkey serum (SL050, Solarbio) and 3% BSA (SRE0096, Sigma) PBST at room temperature for 1 h, followed by overnight incubation in 4 °C with the antibodies listed in the Additional file 1: Table S1. After PBS rinses, sections were incubated with fluorophore conjugated secondary antibodies (Additional file 1: Table S1) for 90 min at room temperature. Fluorescent image acquisition was performed with a Nikon A1 confocal microscope using 10× and 20 × objective lens. Images were analyzed using FIJI ImageJ software (version 1.52p, ImageJ, NIH, USA). Antibody data are provided in the Additional file 1: Table S1.

### Cholera toxin subunit B-Alexa Fluor 488 (CTB–488) injection

100 μg of CTB–488 (C-34775, Thermo Fisher) was dissolved in 100 μl of PBS to the solutions of 1 mg/ml. Aliquots were stored at – 80 °C. Aliquots were thawed and kept on ice before application. A 1 μl total dosage of CTB–488 was injected into the unilateral WIM of mice by 3 injection sites as BoNT/A injection mentioned above. Brains were harvested 10 days later. In addition, the same concentration of CTB-488-mixed BoNT/A was injected as above [36].

### Viral vectors and stereotactic injection

Mice were anesthetized followed by viruses injected into the left WIM as BoNT/A injection mentioned above or into targeted brain nuclei using stereotaxic equipment (68030, Ruiwode Life Science). Viral aliquots of 5 μl were stored at – 80 °C. These aliquots were thawed and kept on ice before injections. Viral data are provided in Additional file 1: Table S1.

For retrograde polytranssynaptic tracing of unilateral WIM, 1 μl PRV-CAG-EGFP was injected into the left WIM at three different sites (3 μl of total dosage) mentioned for BoNT/A injection above after the truncation of the infraorbital nerve of trigeminal ganglion. Brains were harvested every 12 h separately from 24 to 96 h post virus infection.

For anterograde tracing, 100 nl of recombinant adeno-associated virus vector rAAV-hSyn-mGFP-2A-Synaptophysin-mRuby was injected into one side of vlPAG (AP, − 4.72 mm from bregma; ML, − 0.5 mm; DV, − 2.6 mm from the brain surface). Mice were sacrificed 3 weeks later.

To retrogradely manipulate the wFMNs–projecting vlPAG excitatory neurons, 200 nl of monosynaptic retrograde transport virus expressing Cre recombinase with CaMKIIα promotor (rAAV-retro-CaMKIIα-EGFP-Cre) was injected into the wFMNs location in the left lFN (AP, − 6.0 mm from bregma; ML, − 1.25 mm; DV, − 5.8 mm from the brain surface). Meanwhile, 200 nl of Cre-dependent hM4Di or hM3Dq-containing virus that expressed mCherry or a control virus that expressed only mCherry was injected into the ipsilateral vlPAG as mentioned above. Mice were resuscitated on a heating pad after the surgery and then returned to their home cages for a 2-week recovery and for virus expression before the next procedure.

### Clozapine-N-oxide (CNO) administration

CNO (C0832, Sigma) was dissolved by sterile saline to the concentration of 5 mM as stock solution. The stock solution was then diluted to the concentration of 10 μM for working solution, and 1 mg/kg of CNO was *i.p.* injected into the mice. The CNO was administrated for consecutive 21 days to repeatedly inhibit or activate the wFMNs-projecting CaMKII positive neurons of the vlPAG.

### Statistics

Data were analysed using one-way ANOVA followed by Bonferroni’ or Dunnett’s multiple comparisons test to analyse the data from the subgroups. The two-tailed Student’s *t*-test was used for two-group comparisons with normally distributed data. Statistical comparisons were performed using SPSS v22.0 or GraphPad Prism 7 software. Results are presented as means ± SEM, and the statistical methods applied are indicated in the figure legends. Statistical significance was defined as *P* < 0.05. Experimenters were blinded to all subgroup allocation during the experiments.

## Supplementary Information

**Additional file 1: Table S1.** Antibody and viral data.

**Additional file 2: Figure S1**. Flaccid paralysis of unilateral whisker intrinsic musculature injected with BoNT/A 1 day later. (**a**) Normal movement of vibrissae controlled by whisker pad of mice injected with saline. (**b–d**) Dysfunction of vibrissae protracting due to flaccid paralysis of whisker intrinsic musculature induced by three different dosages (3, 10 and 30 U/kg) of BoNT/A injection. **Figure S2. **Related to Figure 1, Locomotor and anxiety-like behaviors of mice performed in OFT are not affected post-BoNT/A unilateral facial injection. (**a**) Schematic of grouping and the OFT testing. (**b**) Mean speed, total distance travelled and duration in the center zone among subgroups of the group receiving BoNT/A injection 6 weeks prior to the restraint end. n = 10 animals from the subgroup of Naïve + Saline, and n = 13 animals from the other four subgroups, respectively. One-way ANOVA followed by Dunnett’s multiple comparisons test comparing each subgroup with the subgroup of CRS mice injected with saline, F _(4,__57)_ = 0.1381, *P* = 0.9675 in mean speed; F _(4,__57)_ = 0.1559, *P* = 0.9595 in total distance; F _(4,__57)_ = 1.06, *P* = 0.3845 in duration in center zone. The *P*-value of Dunnett’s multiple comparisons: Naïve + Saline *vs.* CRS + Saline: *P* = 0.9820, CRS + Saline *vs.* CRS + 3 U/kg: *P* = 0.9999, CRS + Saline *vs. *CRS + 10 U/kg: *P* = 0.9999, CRS + Saline *vs.* CRS + 30 U/kg: *P* = 0.9713 of mean speed; Naïve + Saline *vs.* CRS + Saline: *P* = 0.9890, CRS + Saline *vs.* CRS + 3 U/kg: *P* = 0.8969, CRS + Saline *vs. *CRS + 10 U/kg: *P* = 0.9987, CRS + Saline *vs.* CRS + 30 U/kg: *P* = 0.9397 of total distance; Naïve + Saline *vs.* CRS + Saline: *P* = 0.8624, CRS + Saline *vs.* CRS + 3 U/kg: *P *= 0.3640, CRS + Saline *vs.* CRS + 10 U/kg: *P* = 0.5297, CRS + Saline *vs.* CRS + 30 U/kg: *P* = 0.1871 of duration in center zone. (**c**) Subgroup of CRS mice received 30 U/kg of BoNT/A showed improved duration in the center zone of the OFT than the subgroup of CRS mice injected with saline, though the mean speed and total distance travelled among subgroups were in no significant difference of the group pre-injected with BoNT/A 3 weeks prior to the restraint end. n = 10 animals from the subgroup of Naïve + Saline, and n = 15 animals from the other four subgroups, respectively. one-way ANOVA followed by Dunnett’s multiple comparisons test controlling to the subgroup of mice injected with saline, F _(4,__65)_ = 0.9405, *P* = 0.4463 in mean speed; F _(4,__65)_ = 0.2468, *P* = 0.9106 in total distance; F _(4,__65)_ = 2.856, *P* = 0.0304 in duration in center zone. The *P*-value of Dunnett’s multiple comparisons: Naïve + Saline *vs.* CRS + Saline: *P* = 0.3843, CRS + Saline *vs.* CRS + 3 U/kg: *P* = 0.4148, CRS + Saline *vs.* CRS + 10 U/kg: *P *= 0.5891, CRS + Saline *vs.* CRS + 30 U/kg: *P* = 0.9417 of mean speed; Naïve + Saline *vs. *CRS + Saline: *P* = 0.9976, CRS + Saline *vs. *CRS + 3 U/kg: *P* = 0.7701, CRS + Saline *vs.* CRS + 10 U/kg: *P* = 0.9902, CRS + Saline *vs.* CRS + 30 U/kg: *P* = 0.9999 of total distance; Naïve + Saline *vs. *CRS + Saline: *P* = 0.0113, CRS + Saline* vs.* CRS + 3 U/kg: *P* =0.0786, CRS + Saline *vs. *CRS + 10 U/kg: *P* = 0.3684, CRS + Saline *vs. *CRS + 30 U/kg: *P* = 0.0314 of duration in center zone. (**d**) Mean speed, total distance travelled and duration in center zone among subgroups of the group with pre-injection of BoNT/A 3 days prior to the restraint end. n = 10 animals from the subgroup of Naïve + Saline, and n = 9 animals from the other four subgroups, respectively. One-way ANOVA followed by Dunnett’s multiple comparisons test controlling to the subgroup of mice injected with saline, F _(4,__41)_ = 0.6347, *P* = 0.6407 in mean speed; F _(4,__41)_ = 0.4634, *P* = 0.7621 in total distance; F _(4,__41)_ = 1.496, *P* = 0.2210 in duration in center zone. The *P*-value of Dunnett’s multiple comparisons: Naïve + Saline *vs.* CRS + Saline: *P *= 0.7785, CRS + Saline* vs. *CRS + 3 U/kg: *P* = 0.7375, CRS + Saline *vs. *CRS + 10 U/kg:* P* = 0.3648, CRS + Saline *vs.* CRS + 30 U/kg: *P* = 0.9535 of mean speed; Naïve + Saline *vs.* CRS + Saline: *P* = 0.9878, CRS + Saline* vs.* CRS + 3 U/kg: *P* = 0.9985, CRS + Saline *vs.* CRS + 10 U/kg: *P *= 0.8151, CRS + Saline* vs.* CRS + 30 U/kg: *P* = 0.9991 of total distance; Naïve + Saline *vs.* CRS + Saline: *P* = 0.0755, CRS + Saline *vs. *CRS + 3 U/kg: *P* = 0.8505, CRS + Saline *vs.* CRS + 10 U/kg: *P* = 0.7714, CRS + Saline *vs. *CRS + 30 U/kg: *P* = 0.8700 of duration in center zone. *P < 0.05. **Figure S3. **Related to Figure 2, Absence of BoNT/A-cl.SNAP25_197_ in trigeminal sensory nuclear complex, second- or higher-order nucleus of WIM. (**a–c**) Sp5 and Pr5 of trigeminal nuclear complex and some second-order nucleus of WIM. Scale bar, 200 μm. (**d**) Subnuclei of PAG, dorsal and median raphe nucleus. Right three, magnified view of the left image. Scale bar, 200 μm. (**e**) Central and basal lateral amygdala. Scale bar, 200 μm. (**f**) Hippocampus and lateral habenular nucleus. Scale bar, 250 μm mean grid. (**g**) Medial prefrontal, motor and sensory cortex. Scale bar, 250 μm mean grid. Abbreviation: BLA, Basal lateral amygdala, CPu, Caudate putamen, d/dlPAG, Dorsal/dorsolateral PAG, DRN, Dorsal raphe nucleus, Hipp, Hippocampus, IL infralimbic cortex, IRt, Intermediate reticular nucleus, LHb, Lateral habenular nucleus, l/vlPAG, Lateral/Ventrolateral PAG, M1, Primary motor cortex, M2, Secondary motor cortex, MdD, Dorsal part of medullary reticular nucleus, MRN, Median raphe nucleus, PCRt, Parvicellular reticular nucleus, PrL, Prelimbic cortex, S1, Primary sensory cortex, Sp5C, Caudal part of Sp5, Sp5I, Interpolar part of Sp5, Sp5O, Oral part of Sp5. **Figure S4. **Related to Figure 3, wFMNs are innervated preferentially by vGluT2 terminals of second-order nucleus. (**a**) Absence of CTB-488-labeled neurons in the trigeminal sensory nuclear complex or adjacent regions of FN 10 days later of CTB-488-mixed BoNT/A injection into the unilateral WIM. (**b–d**) Representative image of CTB-488-labeled neurons in lFN 10 days after CTB-488-mixed BoNT/A WIM injection representing wFNMs which were projected by input vGluT2, SerT and vAChT positive terminals, respectively. Scale bar, 20 μm.

## Data Availability

All data generated and analysis value during this study are available from the corresponding author on reasonable request. Additional materials is available at *Cell & Bioscience* online.

## Abbreviations

BDNF: Brain-derived neurotrophic factor
BoNT/A: Botulinum toxin type A
CaMKII: Ca^2+^/calmodulin-dependent protein kinase type II
CeA: Central region of the amygdala
CNS: Central nervous system
ChAT: Choline acetyltransferase
cl.SNAP25_197_: BoNT/A-cleaved SNAP25
CNO: Clozapine-N-oxide
CRS: Chronic restraint stress
fMRI: Functional magnetic resonance imaging
FST: Forced swimming test
GAD67: Glutamate decarboxylase 1
hSyn: Human synapsin promoter
lFN: Lateral facial nucleus
OFT: Open field test
PAG: Periaqueductal grey
PFC: Prefrontal cortex
Pr5: Principle sensory trigeminal nucleus
PRV: Pseudorabies virus
PSD95: Postsynaptic density-95
SNAP25: Synaptosome-associated protein 25
SerT: Serotonin transporter
Sp5: Spinal trigeminal nucleus
SPT: Sucrose preference test
TH: Tyrosine hydroxylase
TpH2: Tryptophan hydroxylase 2
vAChT: Acetylcholine vesicular transporter
vGluT2: Vesicular glutamate transporter 2
vlPAG: Ventrolateral periaqueductal grey
wFMNs: Whisker-innervating facial motoneurons
WIM: Whisker intrinsic musculature
