# Supplementary material for: Neurocircuitry underlying the antidepressant effect of retrograde facial botulinum toxin in mice
Source: Cell Biosci. 2023 Feb 13;13:30. doi: 10.1186/s13578-023-00964-1 (PMC9926702; doi:10.1186/s13578-023-00964-1)
Supplement: Supplementary file 1 — Additional file 1: Table S1. Antibody and viral data. [file 13578_2023_964_MOESM1_ESM.docx]

# Additional file 1: Additional materials

## Antibody and viral data

| **Table S1** | | | |
| --- | --- | --- | --- |
| **Reagent** | | | |
| **Antibody** | **Concentration** | **Sources** | **Identifier** |
| Mouse anti-SNAP25_197_ | 1:200 | RayBiotech | MD-14-0967 |
| Goat anti-SerT | 1:200 | Abcam | ab130130 |
| Mouse anti-vGluT2 | 1:200 | Abcam | ab79157 |
| Goat anti-vAChT | 1:400 | MilliPore | ABN100 |
| Rabbit anti-c-Fos | 1:5000 | Abcam | ab190289 |
| Mouse anti-CaMKⅡ | 1:400 | Abcam | ab22609 |
| Mouse anti-GAD67 | 1:500 | MilliPore | MAB5406 |
| Rabbit anti-SNAP25 | 1:200 | Abcam | ab5666 |
| Mouse anti-ChAT | 1:1000 | Sigma | AMAb91130 |
| Mouse anti-PSD95 | 1:500 | Abcam | ab2723 |
| Rabbit anti-NeuN | 1:500 | Abcam | ab177487 |
| Alexa Fluor 488 goat anti-mouse | 1:400 | Abcam | ab150113 |
| Alexa Fluor 594 goat anti-mouse | 1:400 | Abcam | ab150116 |
| Alexa Fluor 647 goat anti-mouse | 1:400 | Beyotime | A0473 |
| Alexa Fluor 594 goat anti-rabbit | 1:400 | Abcam | ab150080 |
| Alexa Fluor 647 goat anti-rabbit | 1:400 | Beyotime | A0468 |
| Cy3 donkey anti-goat | 1:400 | Beyotime | A0502 |
| **Virus data** | **Titer** | **Sources** | **Identifier** |
| PRV-CAG-EGFP | 2.00E + 0.9 PFU/ml | BrainVTA | P03001 |
| rAAV-hSyn-mGFP-2A-Synaptophysin-mRuby | 4.80E + 12 vg/mL | BrainVTA | PT-1243 |
| rAAV2/2-retro-CaMKⅡ𝛂-EGFP-Cre | ≥ 5E + 12 vg/ml | BrainVTA | PT-0198 |
| rAAV2/9-hSyn-DIO-hM4Di-mCherry | ≥ 5E+12 vg/ml | BrainVTA | PT-0019 |
| rAAV2/9-hSyn-DIO-hM3Dq-mCherry | ≥ 5E+12 vg/ml | BrainVTA | PT-0020 |
| rAAV2/9-hSyn-DIO-mCherry | ≥ 5E+12 vg/ml | BrainVTA | PT-0115 |
