# Supplementary material for: Neurocircuitry underlying the antidepressant effect of retrograde facial botulinum toxin in mice
Source: Cell Biosci. 2023 Feb 13;13:30. doi: 10.1186/s13578-023-00964-1 (PMC9926702; doi:10.1186/s13578-023-00964-1)
Supplement: Supplementary file 2 — Additional file 2: Figure S1. Flaccid paralysis of unilateral whisker intrinsic musculature injected with BoNT/A 1 day later. (a) Normal movement of vibrissae controlled by whisker pad of mice injected with saline. (b–d) Dysfunction of vibrissae protracting due to flaccid paralysis of whisker intrinsic musculature induced by three different dosages (3, 10 and 30 U/kg) of BoNT/A injection. Figure S2. Related to Figure 1, Locomotor and anxiety-like behaviors of mice performed in OFT are not affected post-BoNT/A unilateral facial injection. (a) Schematic of grouping and the OFT testing. (b) Mean speed, total distance travelled and duration in the center zone among subgroups of the group receiving BoNT/A injection 6 weeks prior to the restraint end. n = 10 animals from the subgroup of Naïve + Saline, and n = 13 animals from the other four subgroups, respectively. One-way ANOVA followed by Dunnett’s multiple comparisons test comparing each subgroup with the subgroup of CRS mice injected with saline, F (4, 57) = 0.1381, P = 0.9675 in mean speed; F (4, 57) = 0.1559, P = 0.9595 in total distance; F (4, 57) = 1.06, P = 0.3845 in duration in center zone. The P-value of Dunnett’s multiple comparisons: Naïve + Saline vs. CRS + Saline: P = 0.9820, CRS + Saline vs. CRS + 3 U/kg: P = 0.9999, CRS + Saline vs. CRS + 10 U/kg: P = 0.9999, CRS + Saline vs. CRS + 30 U/kg: P = 0.9713 of mean speed; Naïve + Saline vs. CRS + Saline: P = 0.9890, CRS + Saline vs. CRS + 3 U/kg: P = 0.8969, CRS + Saline vs. CRS + 10 U/kg: P = 0.9987, CRS + Saline vs. CRS + 30 U/kg: P = 0.9397 of total distance; Naïve + Saline vs. CRS + Saline: P = 0.8624, CRS + Saline vs. CRS + 3 U/kg: P = 0.3640, CRS + Saline vs. CRS + 10 U/kg: P = 0.5297, CRS + Saline vs. CRS + 30 U/kg: P = 0.1871 of duration in center zone. (c) Subgroup of CRS mice received 30 U/kg of BoNT/A showed improved duration in the center zone of the OFT than the subgroup of CRS mice injected with saline, though the mean speed and total distance tra [file 13578_2023_964_MOESM2_ESM.docx]

# Additional Figures


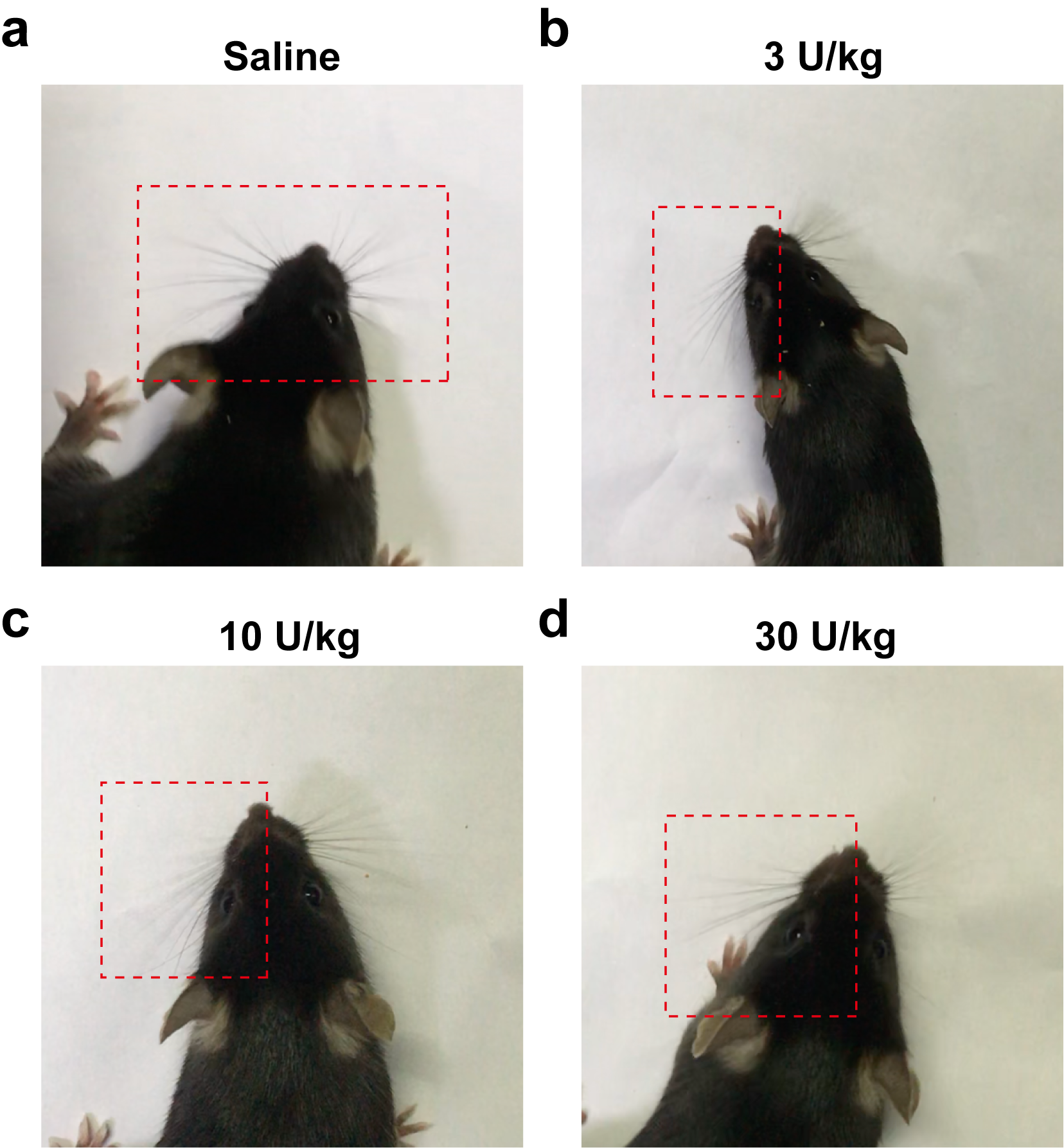


**Figure S1.** **Flaccid paralysis of unilateral whisker intrinsic musculature injected with BoNT/A 1 day later.** (**a**) Normal movement of vibrissae controlled by whisker pad of mice injected with saline. (**b–d**) Dysfunction of vibrissae protracting due to flaccid paralysis of whisker intrinsic musculature induced by three different dosages (3, 10 and 30 U/kg) of BoNT/A injection.


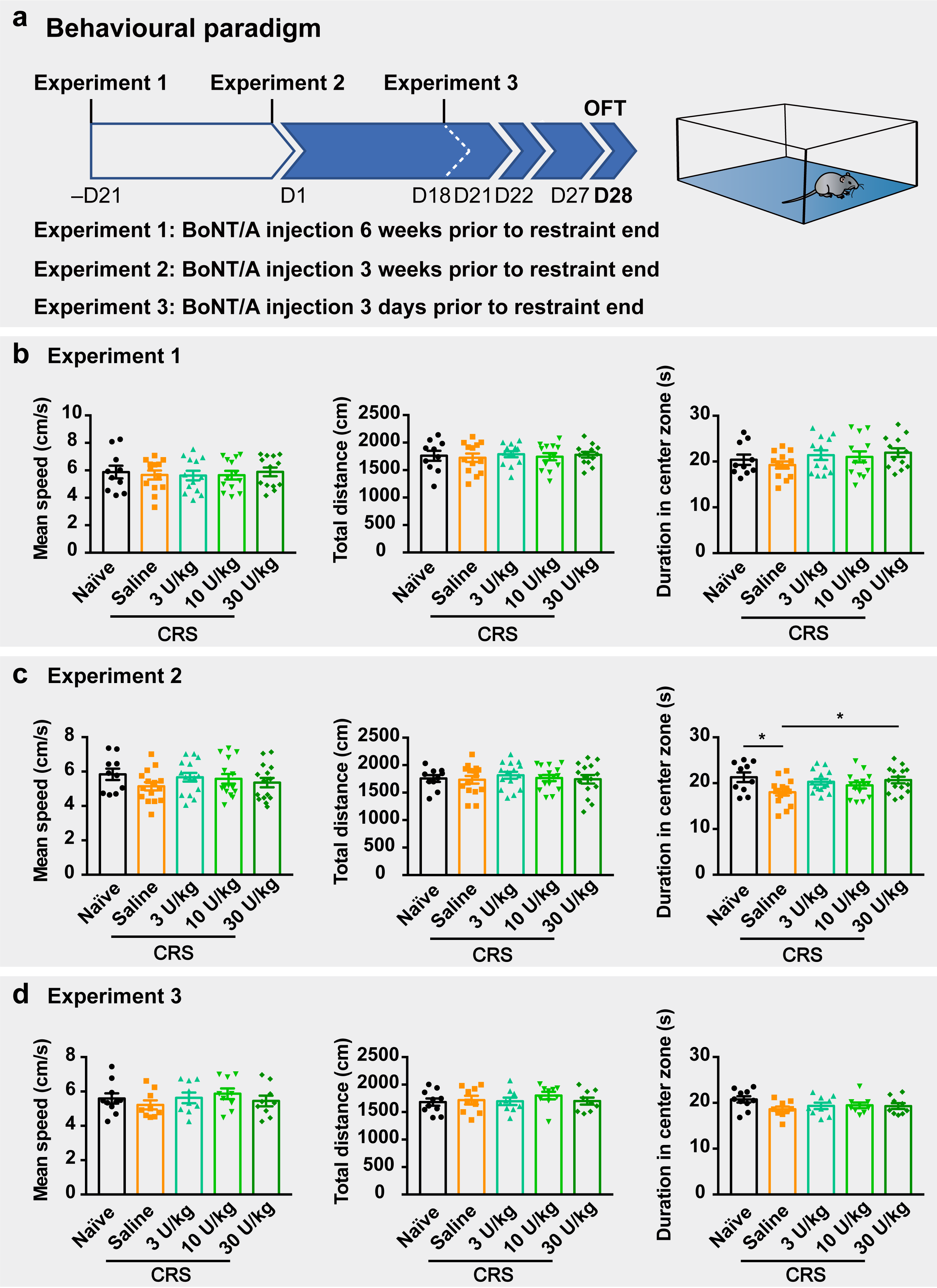


**Figure S2. Related to Figure 1,** **Locomotor and anxiety-like behaviors of mice performed in OFT are not affected post-BoNT/A unilateral facial injection.** (**a**) Schematic of grouping and the OFT testing. (**b**) Mean speed, total distance travelled and duration in the center zone among subgroups of the group receiving BoNT/A injection 6 weeks prior to the restraint end. n = 10 animals from the subgroup of Naïve + Saline, and n = 13 animals from the other four subgroups, respectively. One-way ANOVA followed by Dunnett’s multiple comparisons test comparing each subgroup with the subgroup of CRS mice injected with saline, F _(4, 57)_ = 0.1381, *P* = 0.9675 in mean speed; F _(4, 57)_ = 0.1559, *P* = 0.9595 in total distance; F _(4, 57)_ = 1.06, *P* = 0.3845 in duration in center zone. The *P*-value of Dunnett’s multiple comparisons: Naïve + Saline *vs.* CRS + Saline: *P* = 0.9820, CRS + Saline *vs.* CRS +3 U/kg: *P* = 0.9999, CRS + Saline *vs.* CRS +10 U/kg: *P* = 0.9999, CRS + Saline *vs.* CRS +30 U/kg: *P* = 0.9713 of mean speed in b; Naïve + Saline *vs.* CRS + Saline: *P* = 0.9890, CRS + Saline *vs.* CRS +3 U/kg: *P* = 0.8969, CRS + Saline *vs.* CRS +10 U/kg: *P* = 0.9987, CRS + Saline *vs.* CRS +30 U/kg: *P* = 0.9397 of total distance in b; Naïve + Saline *vs.* CRS + Saline: *P* = 0.8624, CRS + Saline *vs.* CRS +3 U/kg: *P* = 0.3640, CRS + Saline *vs.* CRS +10 U/kg: *P* = 0.5297, CRS + Saline *vs.* CRS +30 U/kg: *P* = 0.1871 of duration in center zone in b. (**c**) Subgroup of CRS mice received 30 U/kg of BoNT/A showed improved duration in the center zone of the OFT than the subgroup of CRS mice injected with saline, though the mean speed and total distance travelled among subgroups were in no significant difference of the group pre-injected with BoNT/A 3 weeks prior to the restraint end. n = 10 animals from the subgroup of Naïve + Saline, and n = 15 animals from the other four subgroups, respectively. one-way ANOVA followed by Dunnett’s multiple comparisons test controlling to the subgroup of mice injected with saline, F _(4, 65)_ = 0.9405, *P* = 0.4463 in mean speed; F _(4, 65)_ = 0.2468, *P* = 0.9106 in total distance; F _(4, 65)_ = 2.856, *P* = 0.0304 in duration in center zone. The *P*-value of Dunnett’s multiple comparisons: Naïve + Saline *vs.* CRS + Saline: *P* = 0.3843, CRS + Saline *vs.* CRS +3 U/kg: *P* = 0.4148, CRS + Saline *vs.* CRS +10 U/kg: *P* = 0.5891, CRS + Saline *vs.* CRS +30 U/kg: *P* = 0.9417 of mean speed in c; Naïve + Saline *vs.* CRS + Saline: *P* = 0.9976, CRS + Saline *vs.* CRS +3 U/kg: *P* = 0.7701, CRS + Saline *vs.* CRS +10 U/kg: *P* = 0.9902, CRS + Saline *vs.* CRS +30 U/kg: *P* = 0.9999 of total distance in c; Naïve + Saline *vs.* CRS + Saline: *P* = 0.0113, CRS + Saline *vs.* CRS +3 U/kg: *P* =0.0786, CRS + Saline *vs.* CRS +10 U/kg: *P* = 0.3684, CRS + Saline *vs.* CRS +30 U/kg: *P* = 0.0314 of duration in center zone in c. (**d**) Mean speed, total distance travelled and duration in center zone among subgroups of the group with pre-injection of BoNT/A 3 days prior to the restraint end. n = 10 animals from the subgroup of Naïve + Saline, and n = 9 animals from the other four subgroups, respectively. One-way ANOVA followed by Dunnett’s multiple comparisons test controlling to the subgroup of mice injected with saline, F _(4, 41)_ = 0.6347, *P* = 0.6407 in mean speed; F _(4, 41)_ = 0.4634, *P* = 0.7621 in total distance; F _(4, 41)_ = 1.496, *P* = 0.2210 in duration in center zone. The *P*-value of Dunnett’s multiple comparisons: Naïve + Saline *vs.* CRS + Saline: *P* = 0.7785, CRS + Saline *vs.* CRS +3 U/kg: *P* = 0.7375, CRS + Saline *vs.* CRS +10 U/kg: *P* = 0.3648, CRS + Saline *vs.* CRS +30 U/kg: *P* = 0.9535 of mean speed in d; Naïve + Saline *vs.* CRS + Saline: *P* = 0.9878, CRS + Saline *vs.* CRS +3 U/kg: *P* = 0.9985, CRS + Saline *vs.* CRS +10 U/kg: *P* = 0.8151, CRS + Saline *vs.* CRS +30 U/kg: *P* = 0.9991 of total distance in d; Naïve + Saline *vs.* CRS + Saline: *P* = 0.0755, CRS + Saline *vs.* CRS +3 U/kg: *P* = 0.8505, CRS + Saline *vs.* CRS +10 U/kg: *P* = 0.7714, CRS + Saline *vs.* CRS +30 U/kg: *P* = 0.8700 of duration in center zone in c. **P* < 0.05.


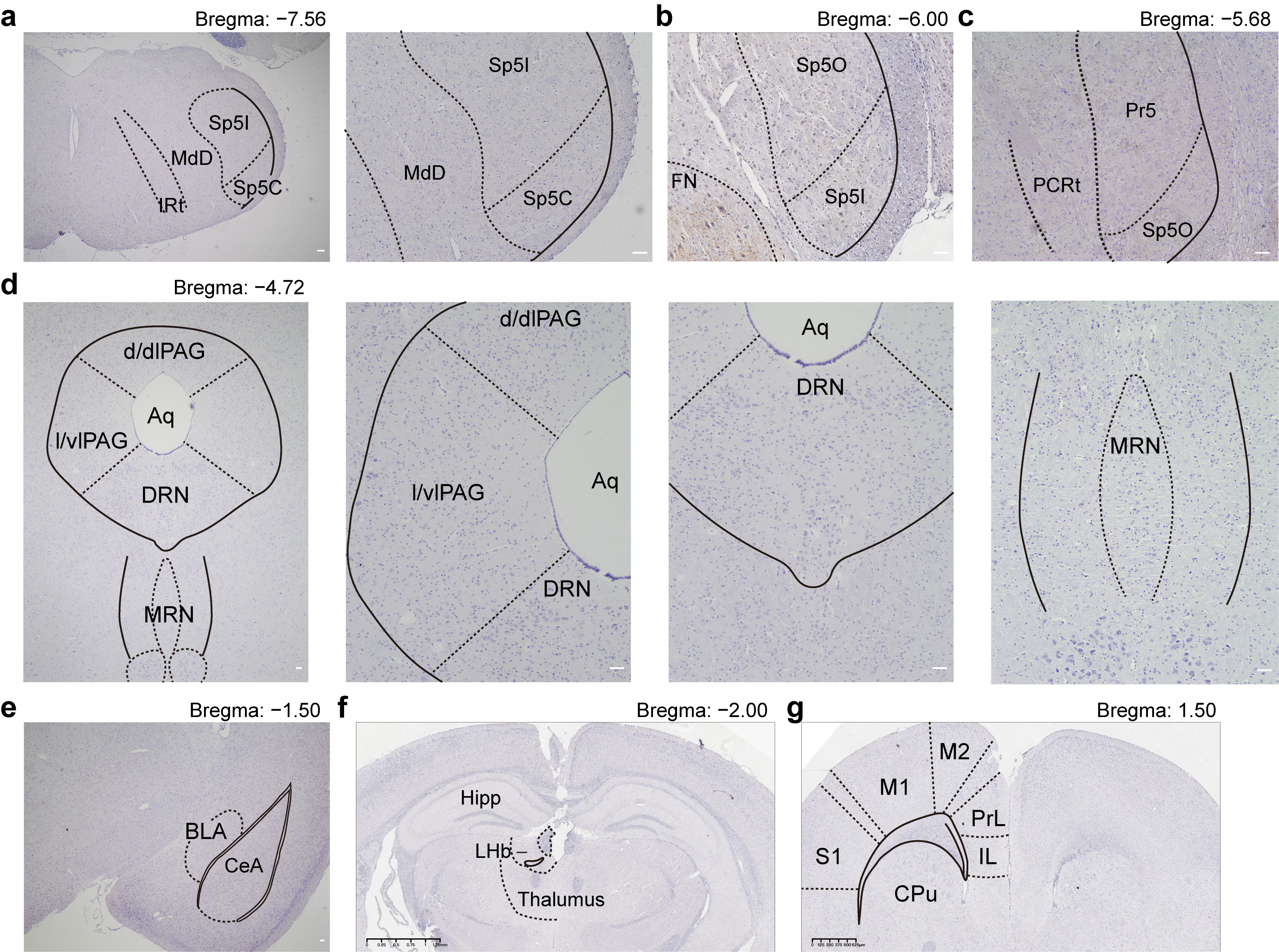


**Figure S3. Related to Figure 2,** **Absence of BoNT/A-cl.SNAP25_197_ in trigeminal sensory nuclear complex, second- or higher-order nucleus of WIM.** (**a–c**) Sp5 and Pr5 of trigeminal nuclear complex and some second-order nucleus of WIM. Scale bar, 200 μm. (**d**) Subnuclei of PAG, dorsal and median raphe nucleus. Right three, magnified view of the left image. Scale bar, 200 μm. (**e**) Central and basal lateral amygdala. Scale bar, 200 μm. (**f**) Hippocampus and lateral habenular nucleus. Scale bar, 250 μm mean grid. (**g**) Medial prefrontal, motor and sensory cortex. Scale bar, 250 μm mean grid. Abbreviation: BLA, Basal lateral amygdala, CPu, Caudate putamen, d/dlPAG, Dorsal/dorsolateral PAG, DRN, Dorsal raphe nucleus, Hipp, Hippocampus, IL infralimbic cortex, IRt, Intermediate reticular nucleus, LHb, lateral habenular nucleus, l/vlPAG, Lateral/Ventrolateral PAG, M1, Primary motor cortex, M2, Secondary motor cortex, MdD, Dorsal part of medullary reticular nucleus, MRN, Median raphe nucleus, PCRt, Parvicellular reticular nucleus, PrL, Prelimbic cortex, S1, Primary sensory cortex, Sp5C, caudal part of Sp5, Sp5I, Interpolar part of Sp5, Sp5O, oral part of Sp5.


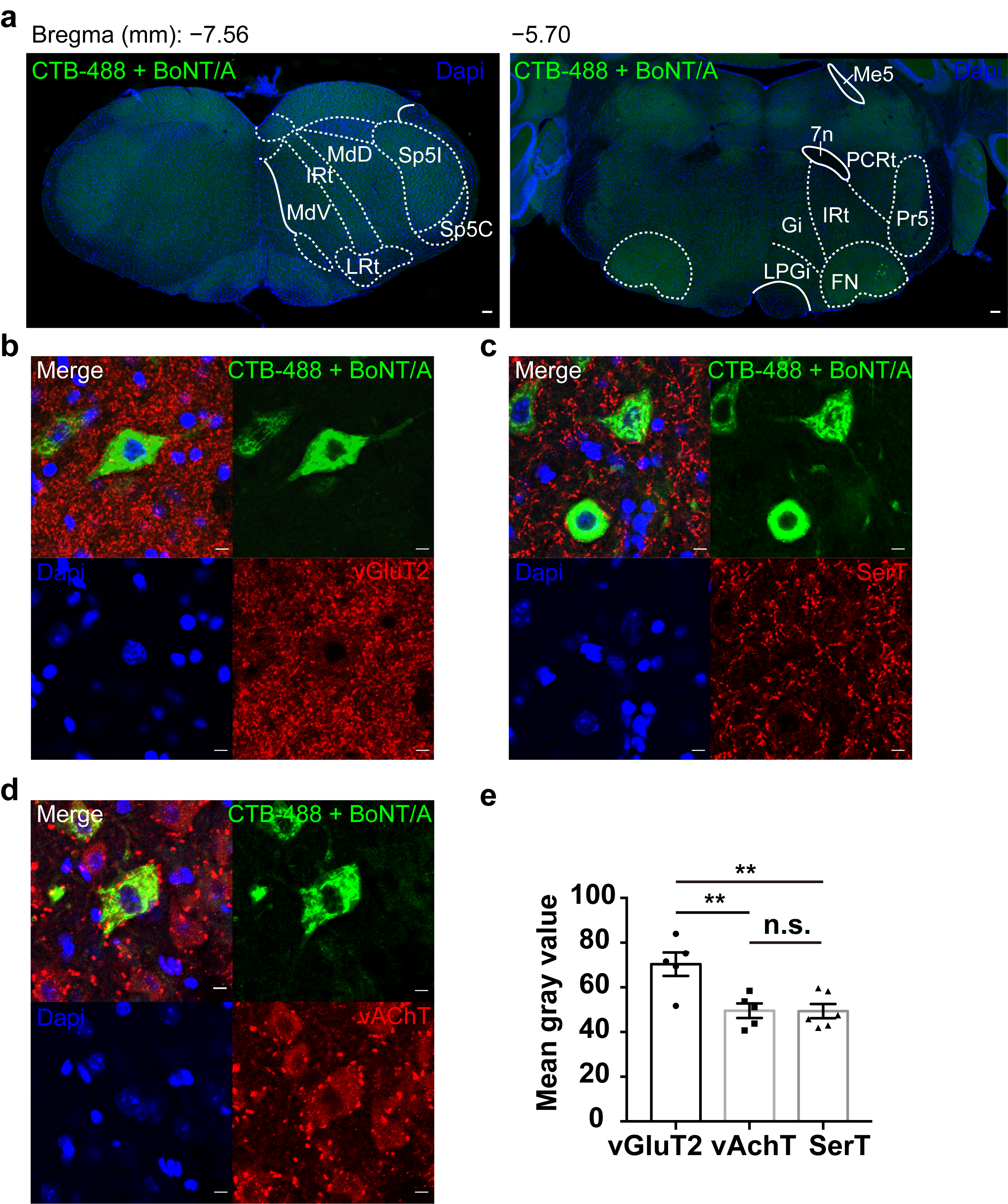


**Figure S4. Related to Figure 3, wFMNs are innervated preferentially by vGluT2 terminals of second-order nucleus.** (**a**) Absence of CTB-488-labeled neurons in the trigeminal sensory nuclear complex or adjacent regions of FN 10 days later of CTB-488-mixed BoNT/A injection into the unilateral WIM. (**b–d**) Representative image of CTB-488-labeled neurons in lFN 10 days after CTB-488-mixed BoNT/A WIM injection representing wFNMs which were projected by input vGluT2, SerT and vAChT positive terminals, respectively. Scale bar, 20 μm. (**e**) Quantificational analysis of mean gray value of terminal signals around CTB-488-labeled neurons among groups. Images were converted into 8-bit gray scale images and the threshold gray value was defined as the background without fluorescence signals. mRuby-labeled signals surrounding each CTB-488-labeled neuron which was exceeding the threshold gray value was to be recorded and the mean gray value per image was obtained. n = 3 mice per group, one-way ANOVA followed by Bonferroni’s multiple comparisons test, F _(2, 13)_ = 9.162, *P* = 0.0033. ***P* < 0.01. Abbreviation: 7n, Facial nerve, Gi, Gigantocellular reticular nucleus, IRt, Intermediate reticular nucleus, LPGi, Lateral paragigantocellular nucleus, LRt, Lateral reticular nucleus, MdD, Dorsal part of medullary reticular nucleus, MdV, medullary reticular nucleus, ventral part, Me5, Mesencephalic trigeminal nucleus, PCRt, Parvicellular reticular nucleus, Sp5C, caudal part of Sp5, Sp5I, Interpolar part of Sp5.


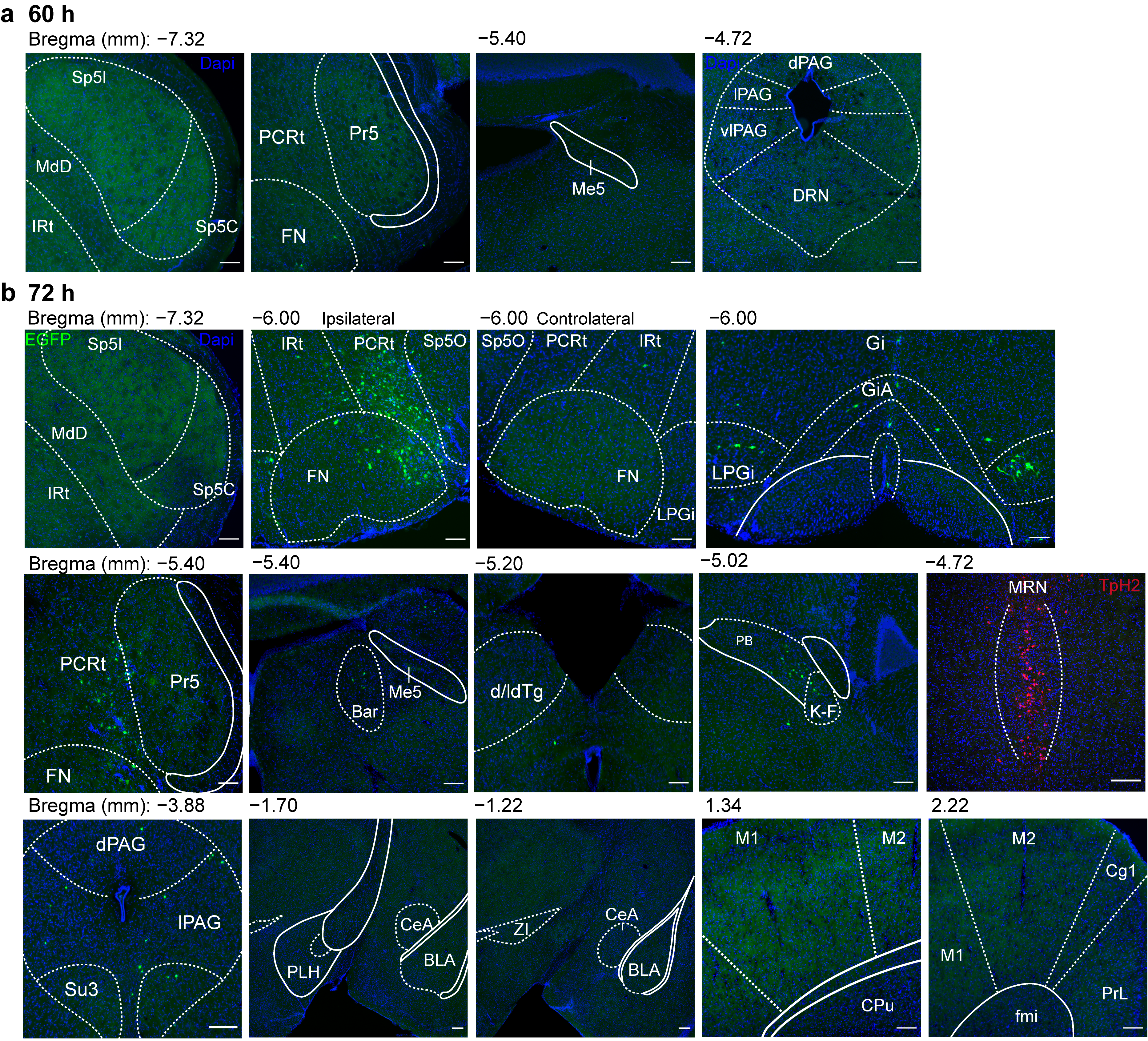


**Figure S5. Related to Figure 4,** **PRV-mediated transsynaptic tracing of WIM premotor neural circuitry.** (**a**) Absence of PRV-infectious neurons in the trigeminal sensory nuclear complex or PAG 60 h later of PRV injection into the WIM. Scale bar, 500 μm. (**b**) Representative images of WIM premotor nuclei labeled by PRV-EGFP, and some higher-order nuclei of WIM were absent from PRV-EGFP 72 h post-PRV WIM injection. Scale bar, 500 μm. Abbreviation: Bar, Barrington’s nucleus, BLA, Basal lateral amygdala, CPu, Caudate putamen, d/dlTg, Dorsal/Dorsolateral tegmental nucleus, dPAG, Dorsal PAG, DRN, Dorsal raphe nucleus, Gi, Gigantocellular reticular nucleus, IRt, Intermediate reticular nucleus, K-F, Klliker-Fuse nucleus, lPAG, Lateral PAG, LPGi, Lateral paragigantocellular nucleus, M1, Primary motor cortex, M2, Secondary motor cortex, MdD, Dorsal part of medullary reticular nucleus, Me5, Mesencephalic trigeminal nucleus, MRN, Median raphe nucleus, PCRt, Parvicellular reticular nucleus, PB, Parabrachial nucleus, PLH, Peduncular part of lateral hypothalamus, PrL, Prelimbic cortex, S1, Primary sensory cortex, Sp5C, caudal part of Sp5, Sp5I, Interpolar part of Sp5, Sp5O, oral part of Sp5, Su3, Supraoculomotor PAG, ZI, Zona incerta.


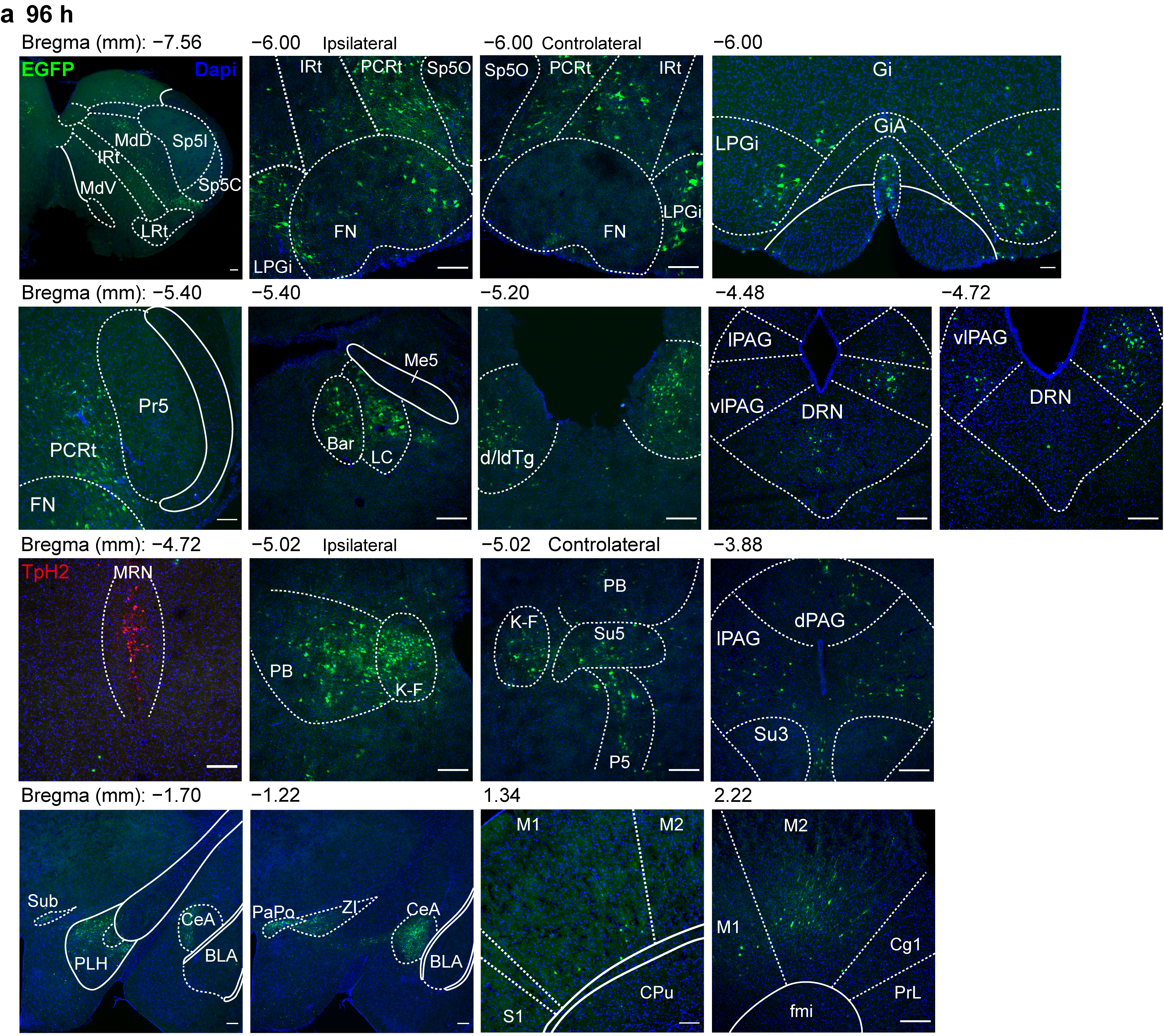


**Figure S6. Related to Figure 4, PRV-mediated transsynaptic tracing higher-nucleus after 96 h of PRV-EGFP unilateral WIM injection.** Scale bar, 500 μm. Abbreviation: Bar, Barrington’s nucleus, BLA, Basal lateral amygdala, Cg1, Cingulate cortex, area 1, CPu, Caudate putamen, d/dlTg, Dorsal/Dorsolateral tegmental nucleus, DRN, Dorsal raphe nucleus, Gi, Gigantocellular reticular nucleus, IRt, Intermediate reticular nucleus, K-F, Klliker-Fuse nucleus, LC, Locus coeruleus, lPAG, Lateral PAG, LPGi, Lateral paragigantocellular nucleus, LRt, Lateral reticular nucleus, M1, Primary motor cortex, M2, Secondary motor cortex, MdD, Dorsal part of medullary reticular nucleus, Me5, Mesencephalic trigeminal nucleus, MRN, Median raphe nucleus, PaPo, Paraventricular hypothalamic nucleus, posterior part, PB, Parabrachial nucleus, PCRt, Parvicellular reticular nucleus, PLH, Peduncular part of lateral hypothalamus, PrL, Prelimbic cortex, S1, Primary sensory cortex, Sp5C, caudal part of Sp5, Sp5I, Interpolar part of Sp5, Sp5O, oral part of Sp5, Su3, Supraoculomotor PAG, Sub, Submedius thalamic nucleus, ZI, Zona incerta.


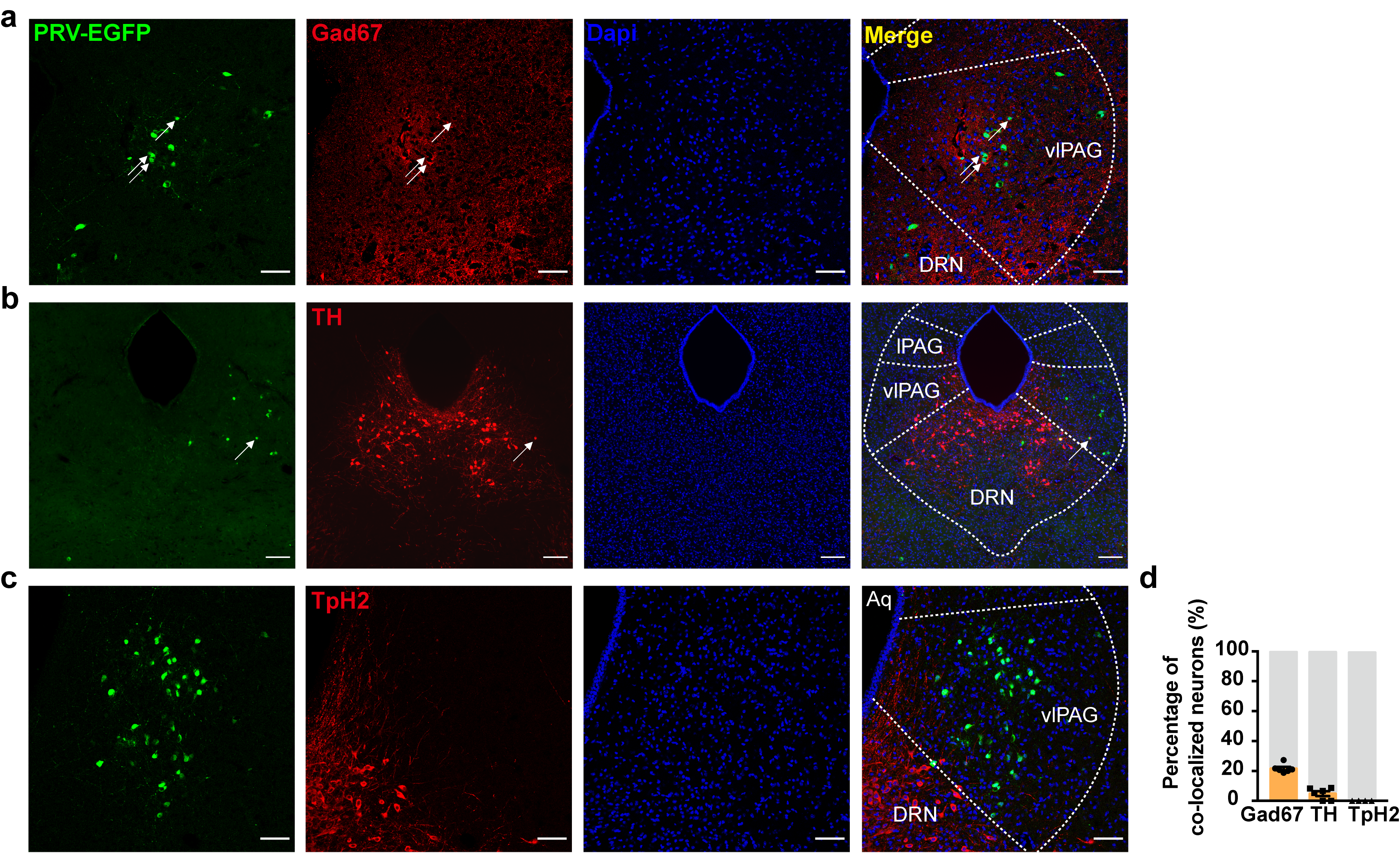


**Figure S7. Related to Figure 4, Neural phenotype characteristics of WIM-premotor neurons in vlPAG.** (**a–c**) Representative images of PRV-EGFP-labeled neurons co-staining with inhibitory neuron marker Gad67, dopaminergic neuron marker TH and serotonergic neuron marker TpH2, respectively. Scale bar, 200 μm. (**d**) Percentage of Gad67, TH and TpH2 positive neurons in PRV-EGFP-labeled neurons, respectively. Abbreviation: DRN, Dorsal raphe nucleus, lPAG, Lateral PAG.


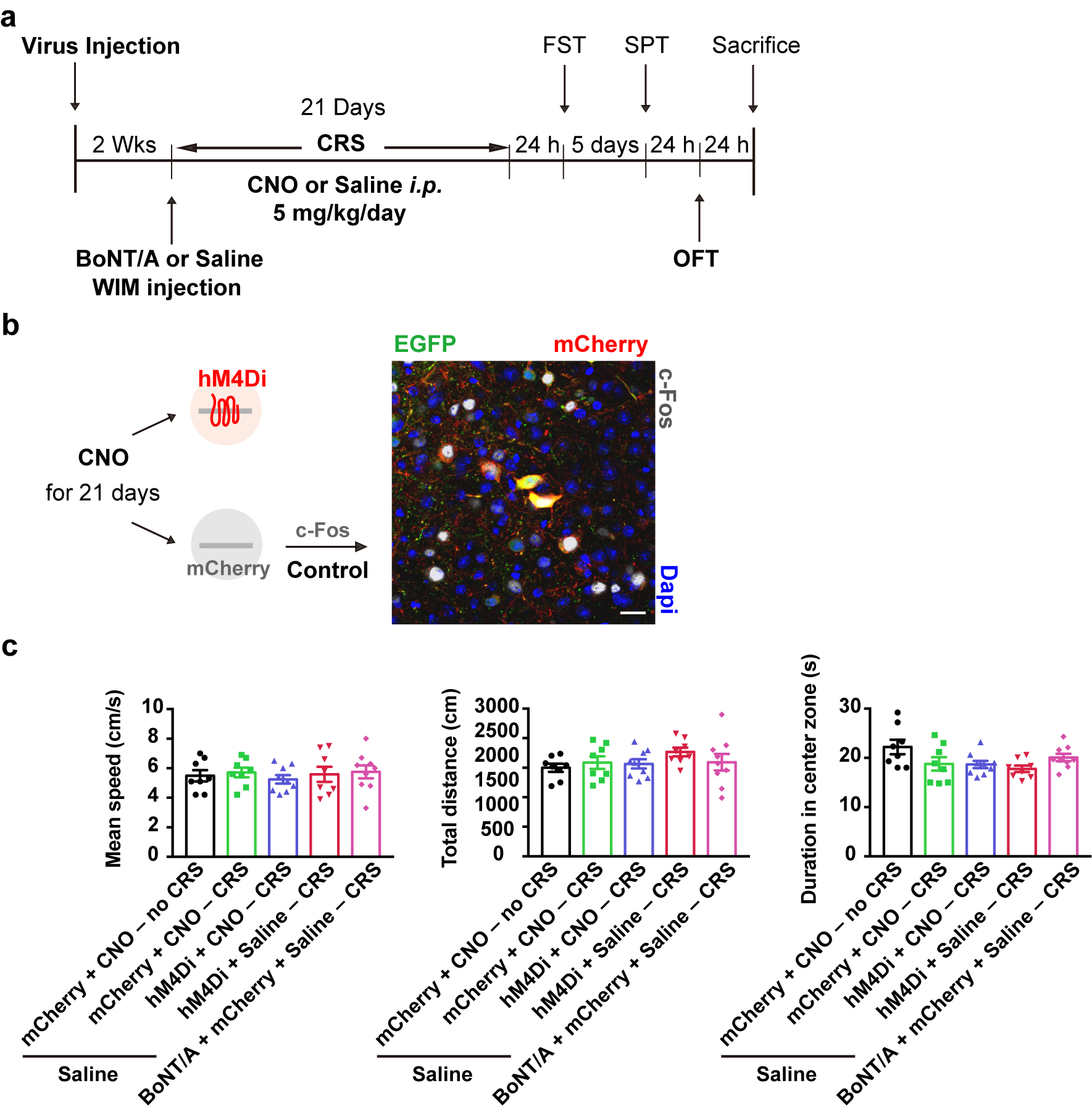


**Figure S8. Related to Figure 6, Inhibition of wFMNs-projecting vlPAG excitatory neurons does not influence the locomotion of CRS mice performed in OFT.** (**a**) Schematic of experimental design. (**b**) Representative image of the vlPAG indicated that *i.p.* injection of CNO induced c-Fos expressions in neurons expressing mCherry of CRS mice. Scale bar, 100 μm. (**c**) Mean speed, total distance travelled and duration in the center zone were in non-alteration performed in the OFT among groups. All mice injected by a rAAV-retro-CaMKⅡα-EGFP-Cre vector into the unilateral lFN at the site of wFMNs. Saline + mCherry + CNO–no CRS, mice that received WIM pre-injection of saline, vlPAG injection of AAV-DIO-mCherry, and *i.p.* injection of CNO without exposure to CRS. Saline + mCherry + CNO–CRS, mice that received WIM pre-injection of saline, vlPAG injection of AAV-DIO-mCherry, and *i.p.* injection of CNO with exposure to CRS. Saline + hM4Di + CNO–CRS, mice that received WIM pre-injection of saline, vlPAG injection of AAV-DIO-hM4Di-mCherry, and *i.p.* injection of CNO with exposure to CRS. Saline + hM4Di + Saline–CRS, mice that received WIM pre-injection of saline, vlPAG injection of AAV-DIO-hM4Di-mCherry, and *i.p.* injection of saline with exposure to CRS. BoNT/A + mCherry + Saline–CRS, mice that received WIM pre-injection of BoNT/A, vlPAG injection of AAV-DIO-mCherry, and *i.p.* injection of saline with exposure to CRS. n = 8 animals from the group of Saline + mCherry + CNO–no CRS, Saline + mCherry + CNO–CRS and Saline + hM4Di + Saline–CRS, n= 9 animals from the group of Saline + hM4Di + CNO–CRS and BoNT/A + mCherry + Saline–CRS. One-way ANOVA followed by Bonferroni’s multiple comparisons test, F _(4, 37)_ = 0.2799, *P* = 0.8891 for mean speed; F _(4, 37)_ = 0.2099, *P* = 0.9313 for total distance; F _(4, 37)_ = 2.689, *P* = 0.0460 for duration in center zone. The *P*-value of Bonferroni’s multiple comparisons: Saline + mCherry + CNO–no CRS *vs.* Saline + mCherry + CNO–CRS: *P* > 0.9999, Saline + mCherry + CNO–no CRS *vs.* Saline + hM4Di + Saline–CRS: *P* > 0.9999, Saline + mCherry + CNO–CRS *vs.* Saline + hM4Di + CNO–CRS: *P* > 0.9999, Saline + mCherry + CNO–CRS *vs.* BoNT/A + mCherry + Saline–CRS: *P* > 0.9999, Saline + hM4Di + CNO–CRS *vs.* Saline + hM4Di + Saline–CRS: *P* > 0.9999, Saline + hM4Di + Saline–CRS *vs.* BoNT/A + mCherry + Saline–CRS: *P* > 0.9999 of mean speed in c; Saline + mCherry + CNO–no CRS *vs.* Saline + mCherry + CNO–CRS: *P* = 0.9850, Saline + mCherry + CNO–no CRS *vs.* Saline + hM4Di + Saline–CRS: *P* = 0.9959, Saline + mCherry + CNO–CRS *vs.* Saline + hM4Di + CNO–CRS: *P* = 0.9867, Saline + mCherry + CNO–CRS *vs.* BoNT/A + mCherry + Saline–CRS: *P* = 0.9003, Saline + hM4Di + CNO–CRS *vs.* Saline + hM4Di + Saline–CRS: *P* = 0.9938, Saline + hM4Di + Saline–CRS *vs.* BoNT/A + mCherry + Saline–CRS: *P* = 0.9990 of total distance in c; Saline + mCherry + CNO–no CRS *vs.* Saline + mCherry + CNO–CRS: *P* = 0.2732, Saline + mCherry + CNO–no CRS *vs.* Saline + hM4Di + Saline–CRS: *P* = 0.1929, Saline + mCherry + CNO–CRS *vs.* Saline + hM4Di + CNO–CRS: *P* > 0.9999, Saline + mCherry + CNO–CRS *vs.* BoNT/A + mCherry + Saline–CRS: *P* > 0.9999, Saline + hM4Di + CNO–CRS *vs.* Saline + hM4Di + Saline–CRS: *P* > 0.9999, Saline + hM4Di + Saline–CRS *vs.* BoNT/A + mCherry + Saline–CRS: *P* > 0.9999 of duration in center zone in c.


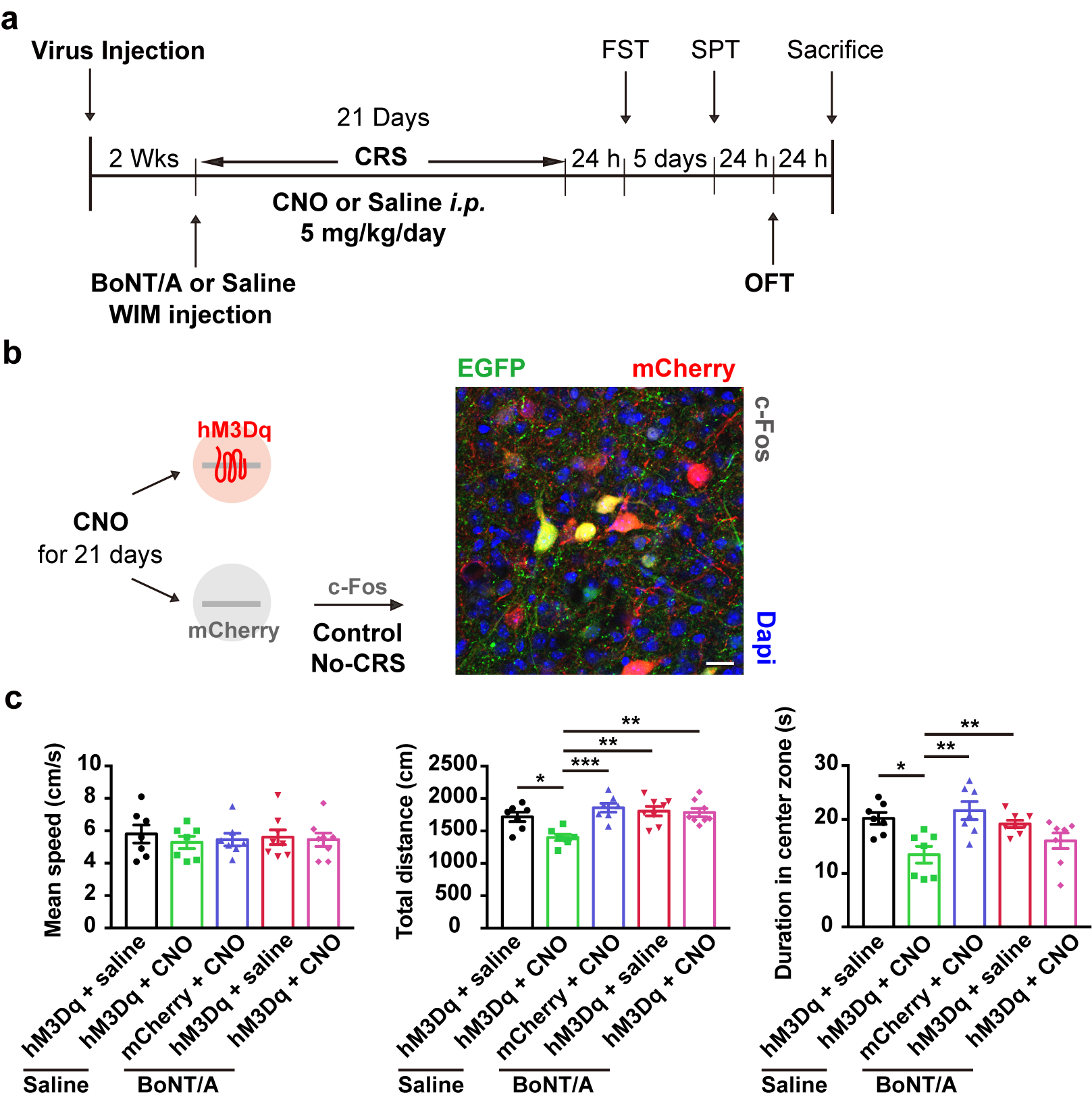


**Figure S9. Related to figure 7,** **Activation of wFMNs-projecting vlPAG excitatory neurons does not influence the locomotion but induces anxiety-like behavior of CRS mice performed in OFT.** (**a**) Schematic of experimental design. (**b**) Representative image of the vlPAG indicated that *i.p*. injection of CNO induced c-Fos expressions in neurons expressing mCherry of mice without restraint stress. Scale bar, 100 μm. (**c**) Mean speed, total distance travelled and duration in the center zone performed in the OFT in different groups. All mice injected by a rAAV-retro-CaMKⅡα-EGFP-Cre vector into unilateral lFN and exposure to CRS. Saline + hM3Dq + Saline, CRS mice that received WIM pre-injection of saline, vlPAG injection of AAV-DIO-hM3Dq-mCherry, and *i.p.* injection of saline. Saline + hM3Dq + CNO, CRS mice that received WIM pre-injection of saline, vlPAG injection of AAV-DIO-hM3Dq-mCherry, and *i.p.* injection of CNO. BoNT/A + mCherry + CNO, CRS mice that received WIM pre-injection of BoNT/A, vlPAG injection of AAV-DIO-mCherry, and *i.p.* injection of CNO. BoNT/A + hM3Dq + Saline, CRS mice that received WIM pre-injection of BoNT/A, vlPAG injection of AAV-DIO-hM3Dq-mCherry, and *i.p.* injection of saline. BoNT/A + hM3Dq + CNO, CRS mice that received WIM pre-injection of BoNT/A, vlPAG injection of AAV-DIO-hM3Dq-mCherry, and *i.p.* injection of CNO. n = 7 animals from the group of Saline + hM3Dq + Saline, Saline + hM3Dq + CNO and BoNT/A + mCherry + CNO, n = 8 animals from the group of BoNT/A + hM3Dq + Saline and BoNT/A + hM3Dq + CNO. One-way ANOVA followed by Bonferroni’s multiple comparisons test, F _(4, 32)_ = 0.1813, *P* = 0.9464 for mean speed; F _(4, 32)_ = 7.214, *P* = 0.0003 for total distance; F _(4, 32)_ = 6.045, *P* = 0.0010 for duration in center zone. The *P*-value of Bonferroni’s multiple comparisons: Saline + hM3Dq + Saline *vs.* Saline + hM3Dq + CNO: *P* > 0.9999, Saline + hM3Dq + Saline *vs.* BoNT/A + mCherry + CNO: *P* > 0.9999, Saline + hM3Dq + Saline *vs.* BoNT/A + hM3Dq + Saline: *P* > 0.9999, Saline + hM3Dq + CNO *vs.* BoNT/A + mCherry + CNO: *P* > 0.9999, Saline + hM3Dq + CNO *vs.* BoNT/A + hM3Dq + Saline: *P* > 0.9999, Saline + hM3Dq + CNO *vs.* BoNT/A + hM3Dq + CNO, *P* > 0.9999, BoNT/A + mCherry + CNO *vs.* BoNT/A + hM3Dq + CNO: *P* > 0.9999, BoNT/A + hM3Dq + Saline *vs.* BoNT/A + hM3Dq + CNO: *P* > 0.9999 of mean speed in c; Saline + hM3Dq + Saline *vs.* Saline + hM3Dq + CNO: *P* = 0253, Saline + hM3Dq + Saline *vs.* BoNT/A + mCherry + CNO: *P* > 0.9999, Saline + hM3Dq + Saline *vs.* BoNT/A + hM3Dq + Saline: *P* > 0.9999, Saline + hM3Dq + CNO *vs.* BoNT/A + mCherry + CNO: *P* = 0.0004, Saline + hM3Dq + CNO *vs.* BoNT/A + hM3Dq + Saline: *P* = 0.0013, Saline + hM3Dq + CNO *vs.* BoNT/A + hM3Dq + CNO, *P* = 0.0026, BoNT/A + mCherry + CNO *vs.* BoNT/A + hM3Dq + CNO: *P* > 0.9999, BoNT/A + hM3Dq + Saline *vs.* BoNT/A + hM3Dq + CNO: *P* > 0.9999 of total distance in c; Saline + hM3Dq + Saline *vs.* Saline + hM3Dq + CNO: *P* = 0.0136, Saline + hM3Dq + Saline *vs.* BoNT/A + mCherry + CNO: *P* > 0.9999, Saline + hM3Dq + Saline *vs.* BoNT/A + hM3Dq + Saline: *P* > 0.9999, Saline + hM3Dq + CNO *vs.* BoNT/A + mCherry + CNO: *P* = 0.0017, Saline + hM3Dq + CNO *vs.* BoNT/A + hM3Dq + Saline: *P* = 0.0433, Saline + hM3Dq + CNO *vs.* BoNT/A + hM3Dq + CNO, *P* > 0.9999, BoNT/A + mCherry + CNO *vs.* BoNT/A + hM3Dq + CNO: *P* = 0.0511, BoNT/A + hM3Dq + Saline *vs.* BoNT/A + hM3Dq + CNO: *P* = 0.9231 of duration in center zone in c. **P* < 0.05, ***P* < 0.01, ****P* < 0.001.
